# Molecular Evolution of the *VP1* Gene in Human Norovirus GII.4 Variants in 1974–2015

**DOI:** 10.3389/fmicb.2017.02399

**Published:** 2017-12-05

**Authors:** Takumi Motoya, Koo Nagasawa, Yuki Matsushima, Noriko Nagata, Akihide Ryo, Tsuyoshi Sekizuka, Akifumi Yamashita, Makoto Kuroda, Yukio Morita, Yoshiyuki Suzuki, Nobuya Sasaki, Kazuhiko Katayama, Hirokazu Kimura

**Affiliations:** ^1^Ibaraki Prefectural Institute of Public Health, Mito, Japan; ^2^Laboratory of Laboratory Animal Science and Medicine, Faculty of Veterinary Medicine, Kitasato University, Towada, Japan; ^3^Infectious Disease Surveillance Center, National Institute of Infectious Diseases, Musashimurayama, Japan; ^4^Division of Virology, Kawasaki City Institute for Public Health, Kawasaki, Japan; ^5^Department of Microbiology, Yokohama City University Graduate School of Medicine, Yokohama, Japan; ^6^Pathogen Genomics Center, National Institute of Infectious Diseases, Musashimurayama, Japan; ^7^Department of Food and Nutrition, Tokyo Kasei University, Itabashi-ku, Japan; ^8^Graduate School of Natural Sciences, Nagoya City University, Nagoya, Japan; ^9^Laboratory of Viral Infection I, Kitasato Institute for Life Sciences, Kitasato University, Minato-ku, Japan; ^10^School of Medical Technology, Faculty of Health Sciences, Gunma Paz University, Takasaki, Japan

**Keywords:** bioinformatics, GII.4, molecular evolution, Norovirus, VP1

## Abstract

Human norovirus (HuNoV) is a leading cause of viral gastroenteritis worldwide, of which GII.4 is the most predominant genotype. Unlike other genotypes, GII.4 has created various variants that escaped from previously acquired immunity of the host and caused repeated epidemics. However, the molecular evolutionary differences among all GII.4 variants, including recently discovered strains, have not been elucidated. Thus, we conducted a series of bioinformatic analyses using numerous, globally collected, full-length GII.4 major capsid (*VP1*) gene sequences (466 strains) to compare the evolutionary patterns among GII.4 variants. The time-scaled phylogenetic tree constructed using the Bayesian Markov chain Monte Carlo (MCMC) method showed that the common ancestor of the GII.4 *VP1* gene diverged from GII.20 in 1840. The GII.4 genotype emerged in 1932, and then formed seven clusters including 14 known variants after 1980. The evolutionary rate of GII.4 strains was estimated to be 7.68 × 10^−3^ substitutions/site/year. The evolutionary rates probably differed among variants as well as domains [protruding 1 (P1), shell, and P2 domains]. The Osaka 2007 variant strains probably contained more nucleotide substitutions than any other variant. Few conformational epitopes were located in the shell and P1 domains, although most were contained in the P2 domain, which, as previously established, is associated with attachment to host factors and antigenicity. We found that positive selection sites for the whole GII.4 genotype existed in the shell and P1 domains, while Den Haag 2006b, New Orleans 2009, and Sydney 2012 variants were under positive selection in the P2 domain. Amino acid substitutions overlapped with putative epitopes or were located around the epitopes in the P2 domain. The effective population sizes of the present strains increased stepwise for Den Haag 2006b, New Orleans 2009, and Sydney 2012 variants. These results suggest that HuNoV GII.4 rapidly evolved in a few decades, created various variants, and altered its evolutionary rate and antigenicity.

## Introduction

Human norovirus (HuNoV) belongs to the genus *Norovirus* in the family *Caliciviridae* and is a major causative agent of acute gastroenteritis in humans worldwide (Green, 2013; Robilotti et al., 2015). The large genetic divergence of HuNoV results in two genogroups and many genotypes (Green, 2013; Robilotti et al., 2015; Vinjé, 2015). Previous epidemiological studies revealed that GI.3, GII.2, GII.3, GII.4, GII.6, GII.12, and GII.17 were prevalent among HuNoV genotypes (Mathijs et al., 2011; Hoa-Tran et al., 2013; Vega et al., 2014a; Kumazaki and Usuku, 2015; Thongprachum et al., 2016). Importantly, GII.4 has caused pandemics of acute gastroenteritis in people of all ages in various countries during the past 10 years (Bull et al., 2006; Siebenga et al., 2009; Robilotti et al., 2015). Notably, multiple GII.4 variants strains having different antigenicity suddenly emerged and caused pandemics of gastroenteritis in several countries (Debbink et al., 2012a,b, 2013; Lindesmith et al., 2012a; Kumazaki and Usuku, 2015; Allen et al., 2016; Qiao et al., 2016; Parra et al., 2017). For example, in the 2006/07 season, the GII.4 variant emerged and spread rapidly in a large number of areas, including Japan, leading to pandemics in people of all ages (Tu et al., 2007; Motomura et al., 2008; Siebenga et al., 2009; Lam et al., 2012). The GII.4 variant 2006b, a representative strain (Den Haag 2006b strain), caused pandemics in five seasons (Hoa-Tran et al., 2013; Kumazaki and Usuku, 2015; Sato et al., 2017). In the 2006/07 season in Japan, it was estimated that over 3 million children and adults presented with acute gastroenteritis due to infections with the GII.4 2006b (Umeda, 2007). Furthermore, in the 2012/13 season, the GII.4 variant 2012, a representative strain (Sydney 2012 strain), also suddenly emerged and caused pandemics of gastroenteritis similar to that caused by the GII.4 2006b strain in the USA, Europe, and Japan (Siebenga et al., 2009; Allen et al., 2014; Vega et al., 2014a; Kumazaki and Usuku, 2015; Vinjé, 2015; Parra et al., 2017).

The HuNoV major capsid (VP1) protein is considered to be closely associated with the infectivity and antigenicity of these strains (Debbink et al., 2012a). Many previous reports suggested that the HuNoV *VP1* gene rapidly evolved, resulting in a large divergence of antigenicity (Allen et al., 2008; Bok et al., 2009; Yang et al., 2010; Debbink et al., 2012a; Zakikhany et al., 2012). Earlier findings also indicated that the rapid evolution of the *VP1* gene in HuNoV is strongly associated with gastroenteritis pandemics caused by HuNoV (Robilotti et al., 2015).

Various bioinformatic techniques for investigating viral genes may contribute to a better understanding of the evolution of the viruses (Andernach et al., 2013; Añez et al., 2013; Sun et al., 2017; Wei and Li, 2017). Of these, phylogenetic analyses using the Bayesian Markov chain Monte Carlo (MCMC) method can characterize evolutionary processes by estimating the date of divergence and evolutionary rates using sequences with a known sample-collection date for various organisms (Drummond and Bouckaert, 2015). Additionally, the Bayesian skyline plot (BSP) allows prediction of the transition of the infectious population size for pathogens (Hall et al., 2016). Conformational epitope analyses using *in silico* approaches also enable us to identify and map binding sites of host antibodies on three-dimensional structures of proteins (Potocnakova et al., 2016). For HuNoV, a large number of studies on viral evolution have been performed using bioinformatics (Giammanco et al., 2014; Kimura et al., 2015; Kobayashi et al., 2015, 2016; Lu et al., 2016; Parra et al., 2017; Siqueira et al., 2017; Tohma et al., 2017). Previous reports described the evolutionary rates of some HuNoV genotypes using the Bayesian MCMC method and linear regression analyses, which suggested differences in such rates among the genotypes (Parra et al., 2017). In addition, the blockade epitopes on the VP1 protein in some HuNoV genotypes were identified by amino acid variation analyses and molecular biological techniques using the virus-like particle (VLP) (Lindesmith et al., 2014). Previous phylogenetic approaches also showed that GII.4 continuously created genetically different clusters, which resulted in the emergence of many variants (Fioretti et al., 2014; Qiao et al., 2016). However, the differences of evolutionary features among all GII.4 variants, including recently discovered strains, have not been comprehensively elucidated (Bok et al., 2009; Siebenga et al., 2010; Qiao et al., 2016). It may be important to understand why GII.4 has frequently produced escape from previously acquired immunity and repeatedly caused large outbreaks. Moreover, a recent study constructed a system for predicting the HuNoV genotype composition in the peak season using a unique algorithm, namely, NOROCAST (Suzuki et al., 2016). Thus, these bioinformatics technologies may contribute to our understanding of the evolution of the HuNoV GII.4 capsid gene, including the changes of antigenicity, for advancing the prevalent prediction systems for HuNoV. In the present study, using various bioinformatic techniques, we compared the molecular evolution of all GII.4 variants strains using globally collected full-length HuNoV GII.4 *VP1* gene sequences.

## Materials and methods

### Strains used in this study

We obtained the full-length nucleotide sequences of HuNoV *VP1* gene from GenBank^1^ in December 2015. The strains employed were classified using norovirus genotyping tool (Kroneman et al., 2011), and sequences of GII.4 genotype were selected. Of these, strains with ambiguous sequences and unknown year of collection were excluded from the dataset. At this point, complete GII.4 *VP1* sequences of approximately 2,000 strains were included in the dataset. However, due to limitation in computing capacity, positive and negative selection analyses could not include sequences with greater than 99.0% identity, as it resulted in over 500 sequences. Thus, the *VP1* sequences with >98.9% identity were omitted from the dataset. Moreover, the dataset was analyzed to detect recombination within the *VP1* gene using RDP4.95 with seven primary exploratory recombination signal detection methods (RDP, GENECONV, BOOTSCAN/RECSCAN, MAXCHI, CHIMAERA, SISCAN, 3SEQ) (Martin et al., 2015). The *p*-value threshold for significance was set as 0.001. Recombinant regions was determined when it was detected by more than four of these methods. However, this dataset contained no recombinant sequences. Finally, 466 strains were included in this study (Figure S1, Table S1). The sequences of the dataset were aligned using MAFFT software (Katoh and Standley, 2013) and were then subjected to bioinformatic analysis as described below.

### Time-scaled phylogenetic tree constructed using the bayesian markov chain monte carlo method

Molecular clock evolutionary dynamics was performed using the Bayesian MCMC method in the BEAST package v2.4.6 (Suchard et al., 2001; Drummond and Rambaut, 2007; Bouckaert et al., 2014). To construct the correct phylogenetic tree (Choudhuri, 2014), we added the nucleotide sequences of all GII genotypes, including porcine NoV GII (GII.11, GII.18, and GII.19) and other HuNoV GII genotypes (18 strains), and an outgroup strain, HuNoV GI genotype (GI.1) into the dataset mentioned above (a total of 488 strains). First, we selected the appropriate substitution model using the jModelTest2 program (Guindon and Gascuel, 2003; Darriba et al., 2012). Next, the best of four clock models (Strict clock, Exponential relaxed clock, Relaxed clock log normal, Random local clock) and two tree prior models (Coalescent constant population, Coalescent exponential population) were determined by the method of path-sampling/stepping stone-sampling marginal-likelihood estimation. Finally, the dataset was analyzed using the strict clock and constant tree prior models. The MCMC chain lengths were 300,000,000 steps with sampling every 20,000 steps using high-performance computers [system: x3850X6, CPU: Intel(R) Xeon (R) CPU E7-8890 v3 @ 2.50GHz, memory: 1TB] with GPU [Tesla K20c; global memory: 5,061 MB, clock speed; 0.71 GHz, number of cores: 2,496]. The analyzed data were evaluated by the effective sample size using Tracer^2^, and values >200 were accepted. The maximum clade credibility tree was constructed after we discarded the first 10 or 15% (“burn-in”) of the trees using the TreeAnnotator v2.4.6 in the BEAST 2 package. The phylogenetic tree was visualized by the FigTree v1.4.0 program. The reliability of branches was supported by 95% highest posterior densities (HPDs). Furthermore, the evolutionary rates of HuNoV GII.4, the variants including more than 10 strains (US95_96, Farmington Hills 2002, Asia 2003, Hunter 2004, Yerseke 2006a, Den Haag 2006b, Osaka 2007, Apeldoorn 2007, New Orleans 2009, and Sydney 2012 variants) and the domains were also estimated using appropriate models selected for each dataset as described above.

### Similarity analysis

Similarities between the aligned nucleotide sequences and the Bristol 1993 variant were visualized using the SimPlot program (Lole et al., 1999). Two strains of Bristol 1993 variant (X76716 and FJ537137) were used as query sequences. The similarity was examined using a window size of 200 nucleotides in length (nt) and a step size of 20 nt in the full-length *VP1* genes.

### Calculation of the phylogenetic distance

Phylogenetic trees were generated from the datasets of all GII.4 strains and each GII.4 variant, including more than three strains, based on the maximum likelihood method in the MEGA6 software. The best substitution models were determined by the jModelTest2. The phylogenetic distances between GII.4 strains were calculated from the constructed phylogenetic trees by the Patristic program (Fourment and Gibbs, 2006).

### Selective pressure analysis

Non-synonymous (dN) and synonymous (dS) substitutions rates at each codon were calculated by the Datamonkey server to estimate the positive and negative selection sites in capsid *VP1* genes of HuNoV GII.4 and each GII.4 variant (Pond and Frost, 2005; Delport et al., 2010). Sites under positive (dN > dS) and negative (dN < dS) selection were determined by three methods (SLAC, FEL, and IFEL) based on a significance level of *p* < 0.05. For SLAC, the two-tail extended binomial distribution was used to culculate the *p*-value. The FEL and IFEL were based on the single degree of freedom likelihood ratio test (chi-squared asymptotic distribution was used) to classify a site as positively or negatively selected.

### Conformational B-cell epitope prediction of HuNoV GII.4

Conformational epitopes on the capsid VP1 protein of each of the GII.4 variants strains (Bristol 1993: X76716, Camberwell 1994: AF145896, US95_96: AF080558, Kaiso 2003: AB303929, Farmington Hills 2002: AY485642, Lanzou 2002: EU310927, Asia 2003: AB220921, Hunter 2004: AY883096, Yerseke 2006a: EF126963, Den Haag 2006b: EF126965, Osaka 2007: AB434770, Apeldoorn 2007: GU270580, New Orleans 2009: GU445325, Sydney 2012: JX459908) were predicted using the following four tools: DiscoTope 2.0 (Kringelum et al., 2012), BEPro (Sweredoski and Baldi, 2008), EPCES (Liang et al., 2007), and EPSVR (Liang et al., 2010). Cut-off values of epitopes were set at −3.7 (DiscoTope 2.0), 1.3 (BEPro), and 70 (EPCES, EPSVR). Consensus sites in all of the four tools and regions with close residues over two of the sites on the VP1 dimer structures were determined as conformational epitopes.

### Mapping of positive selection sites, predicted epitopes, and amino-acid substitutions on the three-dimensional structure of HuNoV GII.4 VP1 proteins

VP1 dimer structural models of each GII.4 variant were constructed using MODELLER v9.15 (Webb and Sali, 2014). The templates for homology modeling were based on the crystal structures of four strains (PDB ID: 1IHM, 4X07, 4OP7, and 4OPS). The capsid structure of GI (PDB ID: 1IHM) was used as a template for the construction of a shell domain in whole VP1 structures. Amino acid sequences of five templates and the targeted strains were aligned by MAFFTash (Katoh et al., 2002; Standley et al., 2007). The constructed models were minimized based on GROMOS96 (van Gunsteren et al., 1996), implemented by Swiss PDB Viewer v4.1 (Guex and Peitsch, 1997), and evaluated by Ramachandran plots through RAMPAGE server (Lovell et al., 2003). The final models were modified and colored by Chimera v1.11.2 (Pettersen et al., 2004). The positive selection sites, conformational epitopes of each variant, and substitution sites of the other variants to the Bristol 1993 strain were mapped on the structure.

### Bayesian skyline plot analysis

The genealogical population size of HuNoV GII.4 strains was estimated by BSP inference using BEAST v2.4.6. Appropriate substitution and clock models were selected as described above. Yerseke 2006a strains were analyzed in a short period for 1 year since the strains with >98.9% sequence identity were omitted. The analyzed plots were visualized with the 95% HPDs using Tracer.

### Statistical analyses

Statistical analyses were performed by EZR statistical software based on the Kruskal–Wallis test with multiple comparisons using the Holm test for the evolutionary rates and phylogenetic distance, and Fisher's exact test for the distribution of negative selection sites (Kanda, 2013). We obtained sample sizes of 18,000–135,000 for the statistical analyses of the evolutionary rates. The values obtained by dividing the numbers of chain lengths by that of the log parameters were used as sample sizes. For the phylogenetic distance analyses, we acquired 3–11,440 data that exhibited the number of all combinations between GII.4 strains included in the dataset (Mizukoshi et al., 2017). Detailed statistical data are shown in Tables S2–S4. Sample sizes of each group are also presented in **Figures 2**, **4**.

## Results

### Time-scale evolution of the globally collected GII.4 strains

**Figure 1:**
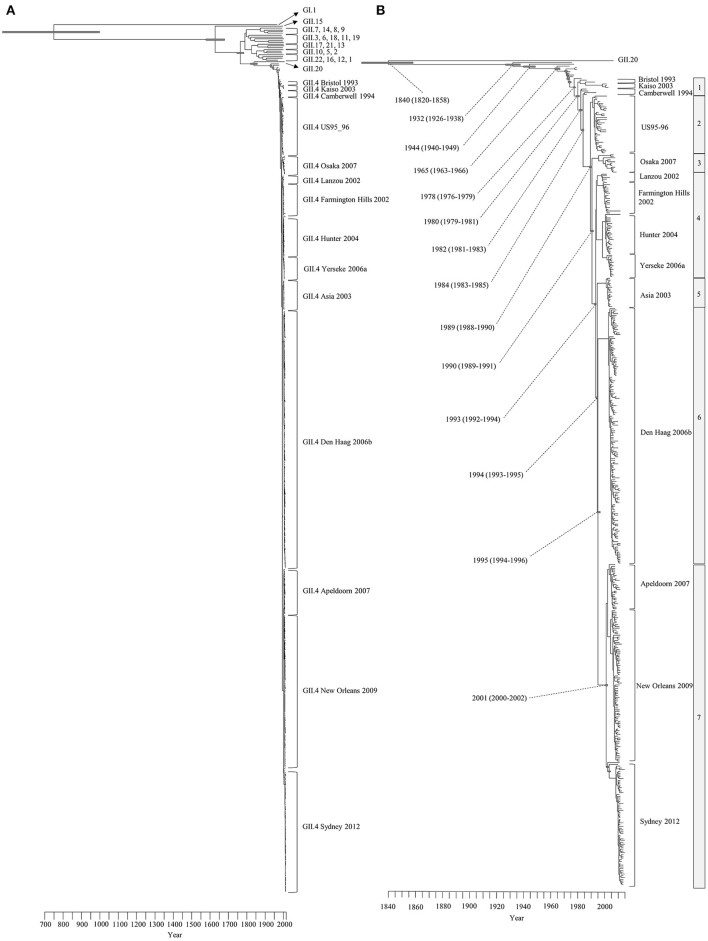
Time-scaled phylogenetic trees of the complete HuNoV capsid *VP1* gene constructed by the Bayesian MCMC method. **(A)** The maximum clade credibility tree with the dataset including HuNoV GI.1 and all GII genotypes; **(B)** Enlarged tree focused on GII.4. Gray bars indicate the 95% highest probability densities for each branch year.

We constructed a time-scale evolutionary phylogenetic tree by the MCMC method using numerous sequences for all GII.4 variants including recent strains. First, a common ancestor of GII.4 strains diverged from GII.20 in 1840 (95% HPDs, 1820–1858). The GII.4 genotype emerged in 1932 (95% HPDs, 1926–1938) and formed seven clusters (seven sublineages) after 1980 (95% HPDs, 1979–1981). Notably, an ancestor of Den Haag 2006b and Sydney 2012 strains, two pandemic GII.4 variants, diverged in 1994 (95% HPDs, 1993–1995) from Asia 2003 strains as a common ancestor. A common ancestor of Apeldoorn 2007, New Orleans 2009, and Sydney 2012 diverged in 2001 (95% HPDs, 2000–2002). Moreover, both pandemic GII.4 variants strains (Den Haag 2006b and Sydney 2012) formed distinct and unique clusters (Figure 1). All divergence years of the clusters are shown in Figure 1B. These results suggest that GII.4 strains rapidly evolved during the past 35–40 years and formed multiple clusters in the phylogenetic tree.

**Figure 2:**
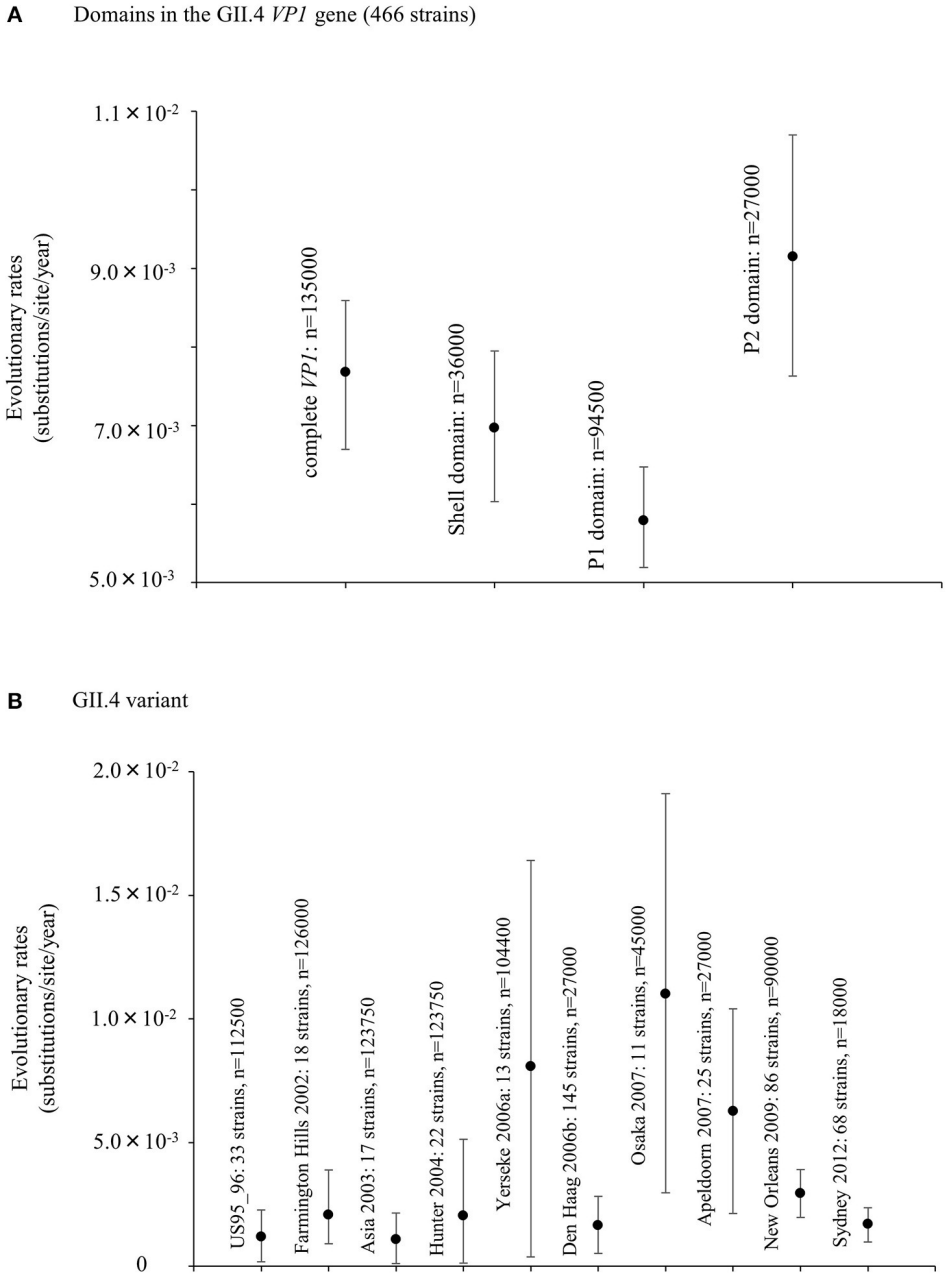
Evolutionary rates of nucleotide sequences in the full-length GII.4 *VP1* gene. **(A)** Evolutionary rates for domains within GII.4 *VP1* gene; **(B)** Evolutionary rates for each GII.4 variant. The y-axis represents the evolutionary rate (substitutions/site/year). Statistical results for multiple comparisons in the domains and the GII.4 variants were shown in Tables S2, S3, respectively.

We also estimated the evolutionary rates of the present GII.4 strains. The evolutionary rate of complete *VP1* gene in the GII.4 strains was estimated to be 7.68 × 10^−3^ substitutions/site/year (95% HPDs, 6.69–8.59 × 10^−3^) (Figure 2A). Moreover, the nucleotide substitution rates were significantly different between the GII.4 variant (Kruskal–Wallis test; *p* < 0.001). The rate of Osaka 2007 variant was higher than among those of other variants (Figure 2B, Table S3). Furthermore, the mean evolutionary rate of the P2 domain (9.15 × 10^−3^ substitutions/site/year) was higher than those of the other domains (shell: 6.97 × 10^−3^, P1 domain: 5.79 × 10^−3^ substitutions/site/year) (Kruskal–Wallis test; *p* < 0.001) (Figure 2A, Table S2).

### Simplot data analyses of the capsid *VP1* gene in the present GII.4 strains

**Figure 3:**
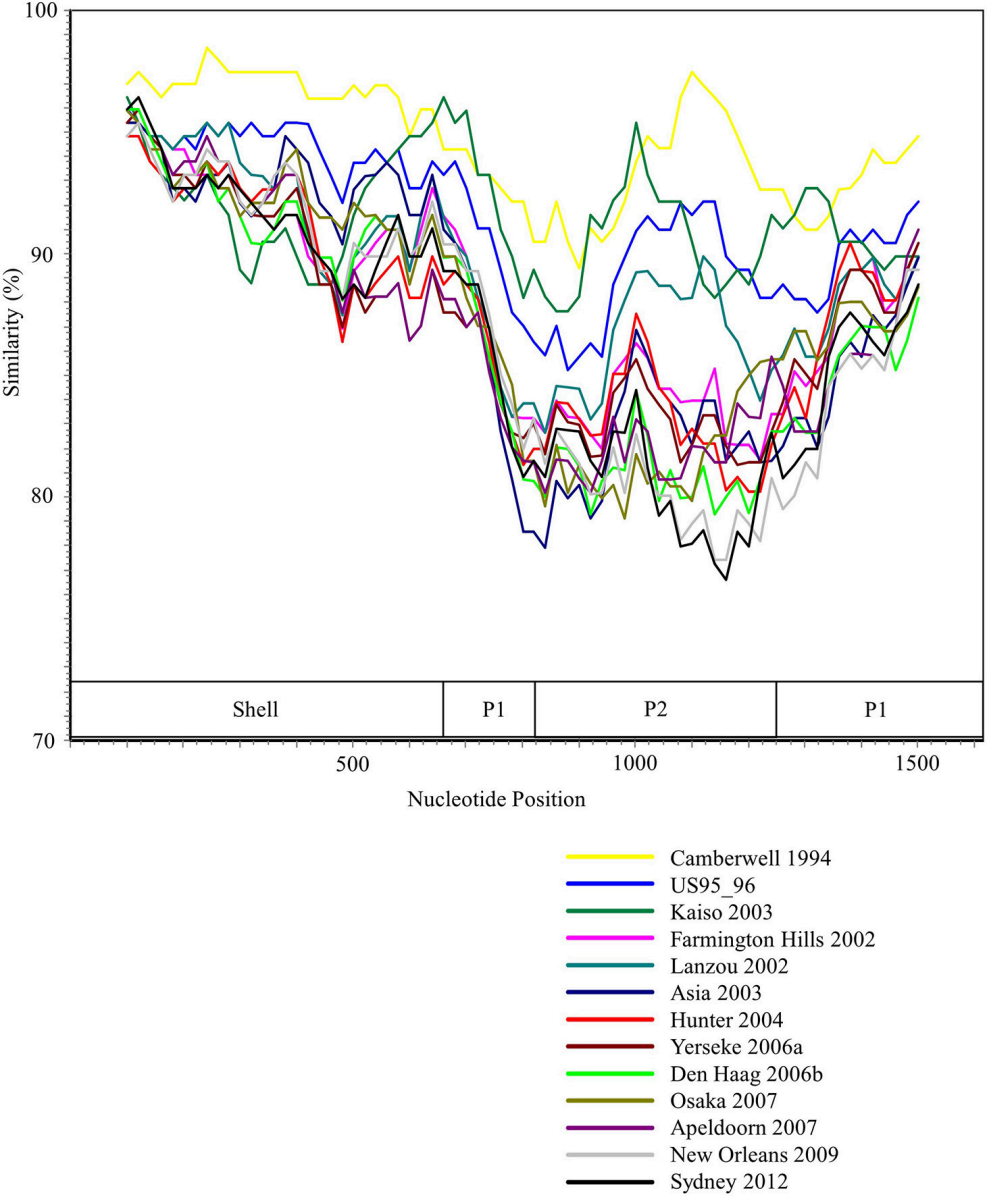
SimPlot analysis of the representative HuNoV GII.4 strains. Each variant's similarity to the Bristol 1993 variant is represented. The positions of the shell, P1, and P2 domains in *VP1* gene are shown below the graph.

We performed the SimPlot analysis based on the full-length capsid gene sequences in the present strains. As illustrated in Figure 3, similarities of 85–100% were found in the shell domain, whereas the P1 and P2 domains were characterized by lower similarities (75–95%). These results indicated that the genetic divergence of the *VP1* gene in the GII.4 strains differed among domains.

### Phylogenetic distances of the capsid *VP1* gene in the GII.4 strains

**Figure 4:**
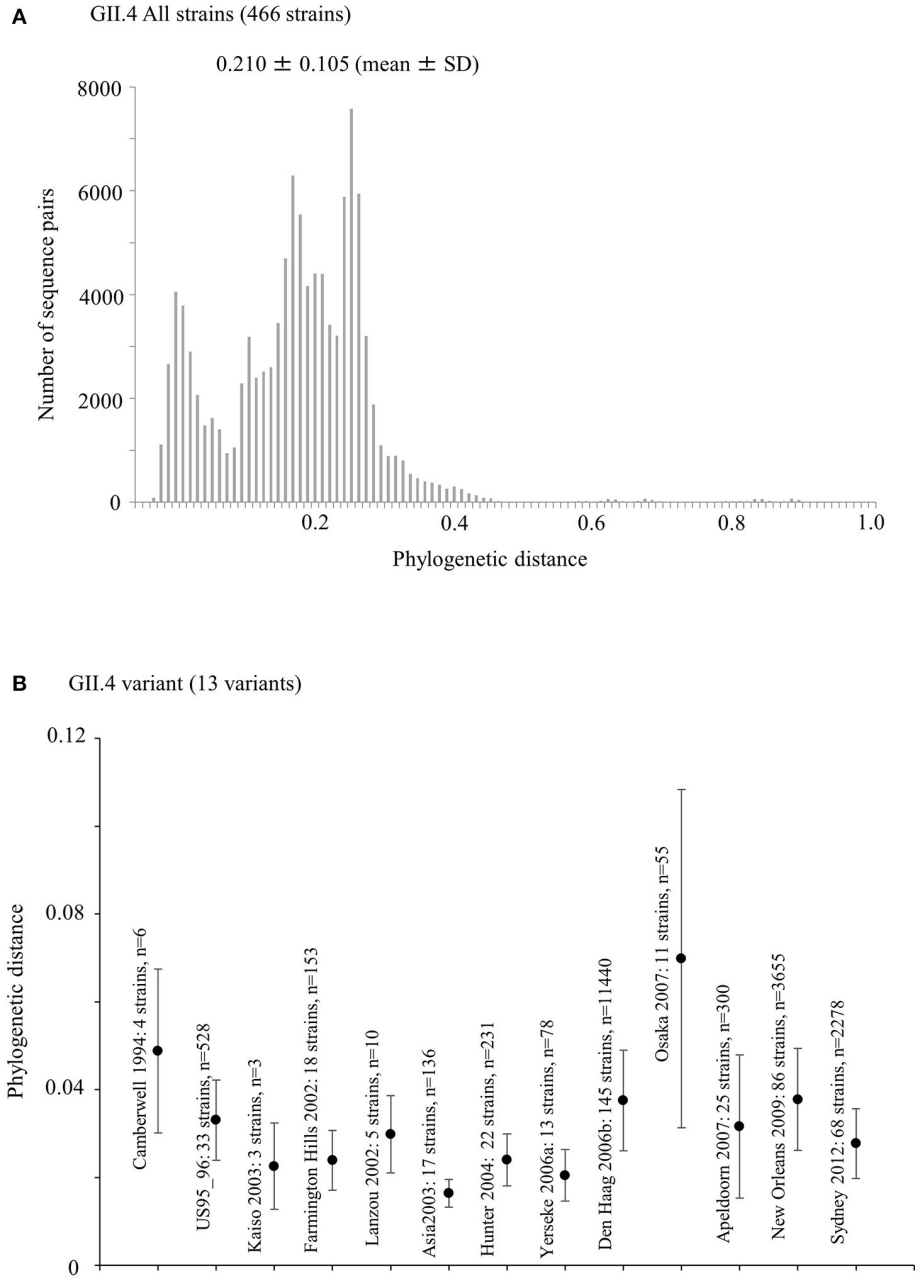
Phylogenetic distance between the nucleotide GII.4 sequences of the full-length *VP1* gene. **(A)** Phylogenetic distance of intra-genotype in HuNoV GII.4 strains. The y-axis represents the number of sequence pairs corresponding to each distance. The x-axis shows phylogenetic distances; **(B)** Phylogenetic distance of intra-variant in HuNoV GII.4 strains. The y-axis indicates phylogenetic distances. The x-axis represents each variant. Data are expressed as mean ± standard deviation. Statistical results for multiple comparisons were shown in Table S4.

We calculated the phylogenetic distance of the *VP1* gene in the GII.4 strains studied here. The phylogenetic distance values of all GII.4 strains were 0.210 ± 0.105 (mean ± standard deviation) (Figure 4A). The mean values of each variant strain were within the range 0.0163–0.0698, and the phylogenetic distances were significantly distinct between GII.4 variants. In particular, the Osaka 2007 variant strains probably included the highest number of nucleotide substitutions, whereas the branches of the strains of the Asia 2003 variant tended to be shorter than those of the other variants (Kruskal–Wallis test; *p* < 0.001) (Figure 4B, Table S4).

### Mapping of conformational epitopes, positive selection sites, and amino-acid substitutions on the VP1 structures of GII.4

**Figure 5:**
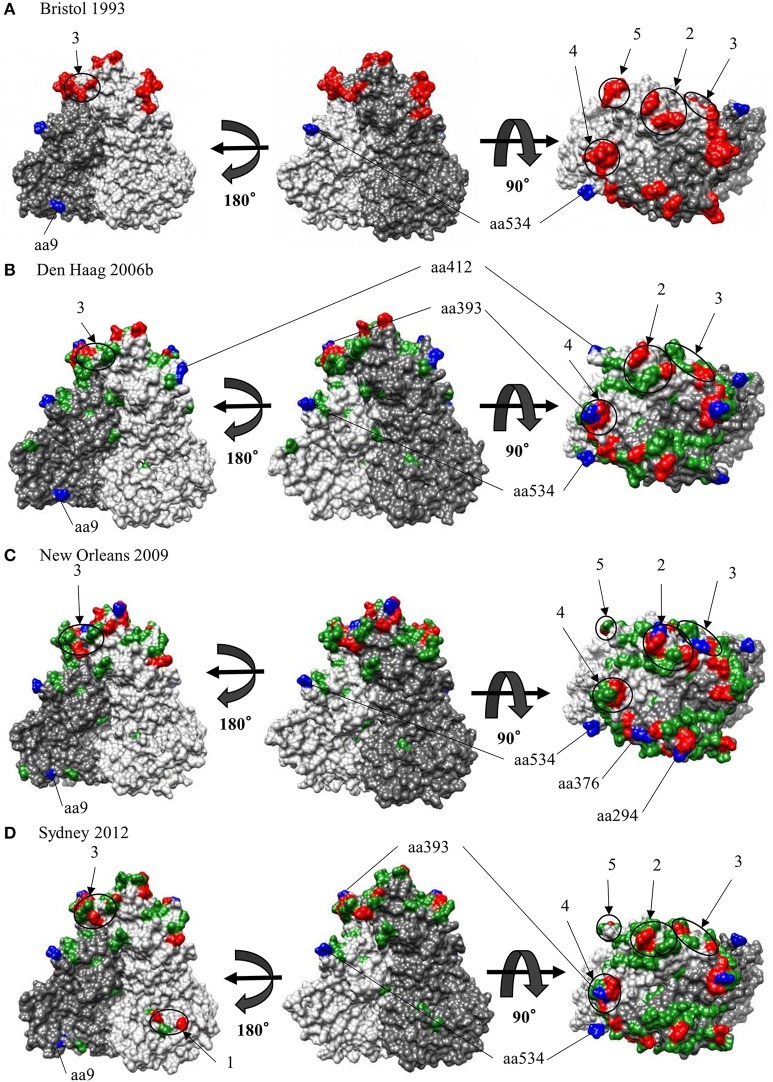
Structural models for the capsid VP1 protein of each HuNoV GII.4 variant. Three-dimensional VP1 dimer structures for the Bristol 1993 **(A)**, the Den Haag 2006b **(B)**, the New Orleans 2009 **(C)** and the Sydney 2012 **(D)** variants are shown. Chains that are composed of the dimer structures are colored in gray (chain A) and dim gray (chain B). Predicted epitopes of each variant are colored in red and circled for regions. Positive selection sites are colored in blue for aa9, aa294, aa376, aa393, aa412, and aa534. Of note, aa6 could not be specified due to lack of structure modeling in N terminus. Amino-acid substitutions of the other variants to a GII.4 Bristol 1993 strain are colored in green.

We also comprehensively predicted the conformational epitope on the whole deduced GII.4 variants VP1 protein and mapped the epitopes, positive selection sites, and amino acid substitutions on the structures of the GII.4 strains to assess their relationships using *in silico* methods (Borley et al., 2013; Yao et al., 2013). Five regions were recognized as conformational epitopes, four of which were located in the exterior surface of the P2 domain and overlapped with the epitope regions identified by previous *in vitro* studies. Moreover, a number of amino-acid substitutions were found on/around the putative epitopes (Table **2**, Figure 5, Figure S2). Positive selection sites for the dataset including all GII.4 strains were predicted to be amino acid (aa) 6 (Asn6Ser and Ser6Asn), aa9 (Asn9Ser, Thr, His, Lys, and Ser9Asn), and aa534 (Thr534Ala, Ser, and Ala534Val, Thr) positions, whereas no positive selection site was found in the P2 domain (Table **3**). Moreover, a total of 369 residues were under negative selection, whose rates were significantly higher in the P1 domain than in the shell and P2 domains (*p* < 0.001) (Table **5**). The positive selection sites for the dataset separating each GII.4 variant was estimated to be aa393 (Ser393Gly, Asn, and Gly393Ser) and aa412 (Asn412Asp, Ser, and Asp412Gly) in Den Haag 2006b; aa294 (Pro294Ser, Thr, Ser294Ala, Pro, and Ala294Thr) and aa376 (Glu376Asp, Val, Gln, Asp376Val, Glu, and Val376Asp, Glu, Ile) in New Orleans 2009; and aa393 (Ser393Gly, Asn, Thr, and Gly393Ser) in Sydney 2012 (Table **4**). The Den Haag 2006b variant contained the most negative selection sites (59 sites), whereas seven GII.4 variants (Camberwell 1994, Kaiso 2003, Farmington Hills 2002, Lanzou 2002, Asia 2003, Hunter 2004, and Yerseke 2006a) were not identified as negative selection sites (Table **5**).

### Phylodynamics of the capsid *VP1* gene in the present GII.4 strains

**Figure 6:**
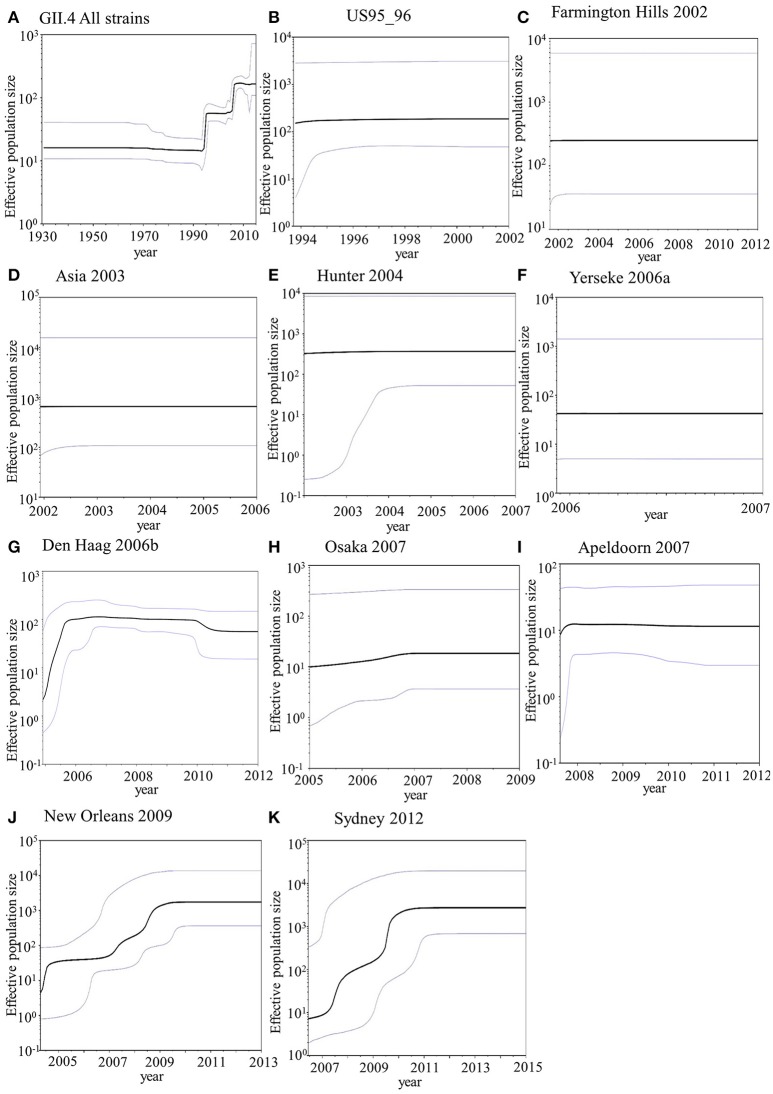
Bayesian skyline plot for *VP1* sequences of HuNoV GII.4. Plots for all GII.4 strains **(A)**, US95_96 variant strains **(B)**, Farmington Hills 2002 variant strains **(C)**, Asia 2003 variant strains **(D)**, Hunter 2004 variant strains **(E)**, Yerseke 2006a variant strains **(F)**, Den Haag 2006 variant strains **(G)**, Osaka 2007 variant strains **(H)**, Apeldoorn 2007 variant strains **(I)**, New Orleans 2009 variant strains **(J)**, and Sydney 2012 variant strains **(K)** are shown. The y-axis represents the effective population size on logarithmic scale, whereas the x-axis denotes the time in years. The solid black line indicates the median posterior value. The intervals with the highest probability densities (95%) are shown by blue lines.

We examined the phylodynamics of human NoV GII.4 strains in the *VP1* gene using the BSP analyses with the parameters shown in Table 1. In the present HuNoV GII.4 strains, the mean effective population size remained constant until approximately 1995 and gradually increased around 1994–1996 and 2005–2006 (Figure 6A). In each GII.4 variant, the mean effective population sizes of Den Haag 2006b, New Orleans 2009, and Sydney 2012 grew in 2005–2007, 2004–2010, and 2006–2011, respectively, whereas no changes of the sizes of the other variants were observed (Figures 6B–K). Moreover, the effective population sizes of all NoV GII.4 strains were estimated to be 10^2^ for approximately 10 years (Figure 6A). These results suggested that restricted sublineages of GII.4 increased the effective population sizes, and the GII.4 strains have adapted to humans for approximately 10 years.

**Table 1:** Parameters for evolutionary rates and Bayesian skyline plot analyses in HuNoV GII.4 and the variants.

**Datasets**	**Number of strains**	**Substitutionmodels**	**Clock models**	**Demographic models**	**Chain length of MCMC**	**Log parameter**
GII.4 all variants (Complete *VP1* gene)	466	GTR+Γ+I	Relaxed clock log normal	Coalescent exponential population	300,000,000	2,000
			Strict clock	Coalescent Bayesian skyline	220,000,000	8,000
GII.4 all variants (Shell domain)	466	HKY+Γ+I	Strict clock	Coalescent exponential population	200,000,000	5,000
GII.4 all variants (P1 domain)	466	SYM+Γ	Relaxed clock log normal	Coalescent exponential population	210,000,000	2,000
GII.4 all variants (P2 domain)	466	GTR+Γ+I	Relaxed clock exponential	Coalescent constant population	150,000,000	5,000
US95_96	33	SYM+Γ+I	Relaxed clock exponential	Coalescent exponential population	250,000,000	2,000
			Relaxed clock exponential	Coalescent Bayesian skyline	100,000,000	2,000
Farmington Hills 2002	18	K80+Γ	Relaxed clock exponential	Coalescent exponential population	1,400,000,000	10,000
			Relaxed clock exponential	Coalescent Bayesian skyline	100,000,000	5,000
Asia 2003	17	K80+Γ	Relaxed clock log normal	Coalescent exponential population	550,000,000	4,000
			Strict clock	Coalescent Bayesian skyline	100,000,000	2,000
Hunter 2004	22	K80+Γ	Relaxed clock exponential	Coalescent exponential population	550,000,000	4,000
			Relaxed clock exponential	Coalescent Bayesian skyline	100,000,000	2,000
Yerseke 2006a	13	TrNef+Γ	Relaxed clock exponential	Coalescent exponential population	1,160,000,000	10,000
			Relaxed clock exponential	Coalescent Bayesian skyline	100,000,000	2,000
Den Haag 2006b	145	HKY+Γ+I	Relaxed clock exponential	Coalescent exponential population	300,000,000	10,000
			Relaxed clock exponential	Coalescent Bayesian skyline	300,000,000	10,000
Osaka 2007	11	TrNef+Γ	Relaxed clock exponential	Coalescent exponential population	100,000,000	2,000
			Relaxed clock exponential	Coalescent Bayesian skyline	100,000,000	2,000
Apeldoorn 2007	25	TrNef+Γ	Relaxed clock exponential	Coalescent exponential population	300,000,000	10,000
			Relaxed clock exponential	Coalescent Bayesian skyline	100,000,000	5,000
New Orleans 2009	86	HKY+Γ+I	Random local clock	Coalescent exponential population	200,000,000	2,000
			Random local clock	Coalescent Bayesian skyline	300,000,000	6,000
Sydney 2012	68	TPM2+Γ+I	Relaxed clock log normal	Coalescent exponential population	100,000,000	5,000
			Random local clock	Coalescent Bayesian skyline	100,000,000	5,000

*Parameters for evolutionary rates and BSP are shown in upper and lower lines, respectively*.

## Discussion

In the present study, we show the molecular evolution of the capsid *VP1* gene in globally collected HuNoV GII.4, including recent strains, during a period of approximately 40 years. Our new findings can be summarized as follows: (1) The evolutionary pattern, including evolutionary rates, phylogenetic distance, and infectious population size, may differ between GII.4 variants. (2) The evolutionary rates and constraints on amino acid mutations are likely distinct between domains within GII.4 *VP1*. (3) There may be few epitopes on the exterior surface of the VP1 structure other than those positions on the P2 domain, as previously identified. (4) Prevalent GII.4 variants specifically undergo selective pressure that leads to new variant that can escape the immune system of the host.

The Bayesian MCMC method is used to characterize evolutionary processes in terms of date of divergence and the rates of evolution for organism genes with high evolutionary rates, such as RNA viruses (Drummond and Bouckaert, 2015). This method has already been used to study molecular evolution not only for noroviruses but also for various other RNA viruses (Añez et al., 2013; Vijaykrishna et al., 2015). In particular, it is important to estimate nucleotide evolutionary rates for HuNoV since previous reports showed that the viruses could evolve with the distinct rates among the genotypes depending on difference of polymerase fidelity and immune pressure for each genotype (Bull et al., 2010). Estimation of evolutionary rates using the method may contribute to predict the frequency of emergence of variants that can escape from previously acquired host immunity in the HuNoV genotypes. However, the results of the method are affected by the sequences included in the dataset. In this study, the time-scaled evolutionary phylogenetic tree showed that the common ancestor of GII.4 and GII.20 dated back to about 175 years ago. Furthermore, during the past 40 years, the GII.4 strains evolved and formed seven clusters including 14 known variants (Figure 1). Next, we estimated the HuNoV GII.4 *VP1* gene evolutionary rate as 7.68 × 10^−3^ substitutions/site/year. Our observations are generally consistent with previous observations overall, with GII.4 having a higher evolutionary rate than other genotypes such as GII.7 (2.3 × 10^−3^ substitutions/site/year), GII.3 (4.16 × 10^−3^ substitutions/site/year), and GII.2 (1.31 × 10^−3^ substitutions/site/year) (Bok et al., 2009; Bull et al., 2010; Siebenga et al., 2010; Boon et al., 2011; Fioretti et al., 2014; Qiao et al., 2016; Mizukoshi et al., 2017; Parra et al., 2017). In addition, we estimated the different evolutionary rates among GII.4 variants. The rates among domains (shell, P1, and P2 domains) in the *VP1* gene also tended to be distinct, with that of the P2 domain being the highest (Figure 2). A previous report showed that GII.4 had replication rates of polymerase that differed among the variants (Bull et al., 2010). In addition, the prevalence of GII.4 differed among the variants (Vinjé, 2015), which may produce differences of herd immunity pressure for each GII.4 variant. We also performed similarity plot analyses in the GII.4 variant strains. Our data showed that the similarities of the shell domains were relatively high (85–100%), followed by a lower level in the P1 domains, and the lowest level in the P2 domains (75–95%), which is consistent with previous reports that showed similarities in the shell, P1, and P2 domains of around >85, >80, and >75%, respectively (Eden et al., 2013; Doerflinger et al., 2017). Next, we calculated the phylogenetic distance among the GII.4 strains. Notably, the values of the intra-variant distance were different. Of them, Osaka 2007 variant strains tended to have high nucleotide variability, while Asia 2003 probably contained fewer nucleotide substitutions than the other variants (Figure 4). These results suggest that GII.4 has uniquely evolved among these variants having different evolutionary rates.

Earlier molecular biological and bioinformatic studies identified or estimated the blocking epitopes in the P2 domain within the GII.4 VP1 structure (Debbink et al., 2012b, 2013; Lindesmith et al., 2012a,b; Parra et al., 2012; Giammanco et al., 2014). These studies were mainly focused on the P2 domain and based on the amino acid variations on the exterior surface in the domain that arose over time. However, other conformational epitopes may also be contained in the shell and P1 domains. Since amino acid sequences of the shell and P1 domains are conserved than those of the P2 domain, it may be possible to use the epitopes of the shell and P1 domains for the development of diagnostic tools for HuNoV, such as immunochromatography kits. Additionally, antibodies associated with antigenicity in positions different from previously identified epitopes may be produced by the B-cell response in the host. To the best of our knowledge, conformational epitope analyses of the whole VP1 structure, including the shell and P1 domains, of various GII.4 variants using mathematical methods have not yet been conducted. The epitopes predicted in our data were close to or overlapped with those positions identified by previous *in vitro* experiments using VLP and monoclonal antibodies (Lindesmith et al., 2012a). Furthermore, our prediction identified few epitopes, including in shell and P1 domains, other than the previously reported positions. This result may suggest difficulty in acquiring antibodies broadly responding to various GII.4 variants that can escape from previously acquired herd immunity. To the best of our knowledge, this is the first observation of this kind in this field. This study suggests the need for additional clarification of the immunology of the GII.4 VP1 protein. However, the present study was performed only *in silico*. To obtain a better understanding of norovirus biology, further studies including *in vitro* epitope-mapping experiments for all antibodies produced by the host should be performed. Next, our analyses revealed that many common epitopes among the GII.4 variants strains were present in the P2 domain. Moreover, a number of substitutions of amino acids were detected in the conformational epitopes (Table 2). Other *in vitro* studies using the HuNoV GII.4 VLP established that the antigenicity changes of the GII.4 were associated with the amino acid substitutions in the P2 domains of GII.4 VP1 proteins (White, 2014). Additionally, previous *in vitro* and bioinformatic studies predicted contact residues specific to antibodies in epitopes A (aa294-298, aa362, 368), B (aa333, aa382), C (aa340, aa376), D (aa393–395), and E (aa407, aa412–413). It has also been confirmed that epitope A is an antigenic region, whereas epitope D is associated with HBGA binding sites. Moreover, epitope C has been predicted to be an evolving epitope and was confirmed using a monoclonal antibody. Epitope E is associated with the potential blockade of Farmington Hills 2002 variant. However, there are no reports on epitope B based on *in vitro* experiments. Previous bioinformatic studies suggested that the five epitopes are associated with the production of antibodies against various GII.4 strains on the exterior surface in the P2 domain (Debbink et al., 2012b, 2013; Lindesmith et al., 2012a,b; Parra et al., 2012; Giammanco et al., 2014; Garaicoechea et al., 2015). Thus, based on our results and those of other researchers, we propose that previously identified epitopes are robustly associated with the antigenicity of HuNoV GII.4.

**Table 2:** Putative conformational epitopes for the capsid VP1 proteins of HuNoV GII.4 variants.

	**Region1**			**Region2**											**Region3**									**Region4**										**Region5**				
	**129**	**171**	**172**	**291**	**293**	**294**	**295**	**296**	**297**	**298**	**368**	**372**	**373**	**374**	**338**	**339**	**340**	**341**	**342**	**343**	**376**	**377**	**378**	**391**	**392**	**393**	**394**	**395**	**396**	**397**	**398**	**443**	**444**	**407**	**411**	**412**	**413**	**414**
Epitope^†^						A		A	A	A	A	A					C				C					D	D	D						E		E	E	
Bristol 1993	P	A	N	T	I	A	G	S	H	D	T	N	N	D	T	R	A	D	G	S	Q	A	G	D	G	D	-	H	H	Q	N	G	Y	N	R	T	G	H
Camberwell 1994	·	S	H	·	·	V	·	·	·	·	·	·	·	·	·	·	·	·	·	·	·	T	·	·	·	·	-	·	·	·	·	·	·	·	·	·	·	·
US95_96	·	·	H	·	·	·	·	·	·	·	·	·	·	·	·	·	G	·	·	·	·	T	·	·	·	·	-	N	·	·	·	·	·	·	·	·	·	·
Kaiso 2003	·	S	·	·	·	·	·	·	R	N	S	D	·	·	·	·	·	·	·	·	·	P	·	·	·	·	-	R	·	·	·	·	·	D	·	·	·	·
Farmington Hills 2002	·	S	·	·	·	·	·	T	·	N	N	·	·	·	·	·	G	·	·	·	E	T	·	·	·	N	G	T	·	·	·	·	·	S	·	·	·	·
Lanzou 2002	·	S	·	·	·	·	·	T	·	·	·	·	·	·	·	·	G	·	·	·	E	T	·	·	·	N	S	A	·	·	·	·	·	N	·	·	·	·
Asia 2003	·	S	·	I	·	P	·	T	R	T	A	D	·	·	·	K	G	·	·	·	E	T	·	·	·	S	S	A	·	R	·	·	·	D	·	·	V	·
Hunter 2004	·	S	·	·	·	·	·	T	Q	N	S	S	·	·	·	·	R	·	·	·	E	T	·	·	·	S	T	T	·	·	·	·	·	D	·	D	S	·
Yerseke 2006a	·	S	·	·	·	·	·	T	Q	E	S	S	·	·	·	·	R	·	·	·	E	T	·	·	·	S	T	T	·	·	·	·	·	D	·	D	S	·
Den Haag 2006b	·	S	·	·	·	·	·	·	R	N	S	E	·	·	·	K	G	·	·	·	E	T	H	·	·	S	T	T	·	R	·	·	·	S	·	N	V	·
Osaka 2007	·	S	·	·	·	·	·	·	R	N	A	D	·	·	·	·	S	·	·	·	E	S	·	·	·	S	T	T	·	R	·	·	·	·	·	·	·	·
Apeldoorn 2007	·	S	·	·	·	·	·	·	R	N	A	D	·	·	·	·	·	·	·	·	D	·	N	·	·	N	T	A	·	R	·	·	·	S	·	N	S	·
New Orleans 2009	·	S	·	·	·	P	·	·	R	N	A	D	·	·	·	·	T	N	·	·	E	T	N	·	·	S	T	T	P	R	·	·	·	S	·	N	I	·
Sydney 2012	·	S	·	·	·	T	·	·	R	N	E	D	R	·	·	·	T	·	·	·	E	·	N	·	·	G	T	T	·	R	·	·	·	S	·	N	T	·

*Red colors represent putative epitopes of each HuNoV GII.4 variant in this analysis*.

† *Amino acids corresponding to epitope regions identified by Lindesmith et al. (2012a) were shown*.

We also estimated the selective pressures in the present GII.4 *VP1* gene. The results obtained by the SLAC, FEL, and IFEL methods revealed positive selection for the whole GII.4 genotype at aa6, aa9, and aa534, which are located in the shell and P1 domains, whereas no positive selection sites were identified in the P2 domain (Table 3). However, separating for each GII.4 variant, positive selection sites in three prevalent GII.4 variants strains were predicted in the P2 domain (aa393 and aa412 in Den Haag 2006b, aa294, and aa376 in New Orleans 2009, and aa393 in Sydney 2012) (Table 4). These sites mostly overlapped with the epitopes that were identified by our prediction and previous *in vitro* studies (Table 2). Positive selection implies that non-synonymous substitutions facilitate the survival of organisms, such as escape from the immune response of the host (Kryazhimskiy and Plotkin, 2008). Moreover, a previous report predicted an association with positive selection of efficient RNA translation via secondary RNA structures (Siebenga et al., 2010). These results indicate that the prevalent GII.4 variants specifically undergoes strong selective pressure that may produce immune escape variants due to the numerous infectious populations with herd immunity. However, the importance of the positive selection in the shell and P1 domains requires further investigation by *in vitro* studies. Previous studies also estimated the positive selection sites in the GII.4 *VP1* gene (Lindesmith et al., 2008; Bok et al., 2009; Siebenga et al., 2010; Vega et al., 2014b). For example, using the SLAC method, Bok et al. (2009) predicted six sites of positive selection (aa6, aa9, aa15, aa47, aa395, and aa534). However, the threshold for statistical significance in that study was higher than that in our study (*p* < 0.25). Siebenga et al. (2010) identified eight positive selection sites using FEL and IFEL (aa6, aa9, aa47, aa352, aa372, aa395, aa407, and aa534), which is partially consistent with our results. The partial discrepancy between our data and those in previous reports might have resulted from the difference in the datasets between studies. Moreover, our results revealed many sites under negative selection. In each domain, negative selection sites accounted for approximately 60–80%. The proportion of negative selection sites tended to be distinct between the domains in the *VP1* gene (Table 5). However, low numbers of strains may result in no or few negative selection sites in some GII.4 variants. Negative selection exhibits non-synonymous substitutions reduce the survival of organisms. Namely, the sites should preserve synonymous substitutions to maintain the survival (Suzuki, 2004). These results suggest that a number of mutations in the *VP1* gene may be under negative and neutral selection.

**Table 3:** Positive selection sites in the present strains of HuNoV GII.4.

**Amino acid changes**	**SLAC**	**FEL**	**IFEL**
Asn6Ser	°	°	°
Ser6Asn			
Asn9Ser, Thr, His, Lys	°	°	°
Ser9Asn			
Asn17Ser, His, Thr		°	
Ala294Val, Thr, Pro, Gly	°		
Val294Gly, Ala			
Thr294Ala, Ile, Ser			
Pro294Ser, Thr			
Ser294Pro, Ala			
Gly294Arg			
Tyr352Ser, Arg, Phe, Leu			°
Ser352Tyr, Leu			
Leu352Phe			
Thr368Ser, Ala, Asn			°
Ala368Val, Ser, Thr, Asp			
Ser368Gly, Asn, Arg			
Gly368Ala, Ser			
Asn368Glu, Asp, Ser			
Glu368Ala, Gly			
Val368Phe			
Glu376Gln, Asp, Val			°
Gln376Glu, Asn			
Asp376Glu, Val, Gly			
Val376Glu, Ile			
Gly393Ser, Asn, Asp		°	°
Asn393Asp, Ser, Gly			
Ser393Asn, Gly, Thr, Ala			
Asp393Asn, Gly, Glu			
Thr395Asn, Ala		°	°
Asn395His, Thr			
His395Arg, Asp, Pro			
Ala395Thr			
Val413Gly, Ala, Ile			°
Gly413Ser, Val, Asn			
Ser413Thr, Asn, Gly			
Thr413Ser, Ile, Ala			
Ile413Thr, Val			
Ser494Thr, Pro, Ala	°		
Thr494Ala			
Thr534Ala, Ser	°	°	°
Ala534Val, Thr			
Total	5	6	9

*Mean dN/dS = 0.130 (95%CI = 0.124–0.136)*.

*p-value < 0.05*.

**Table 4:** Positive selection sites in HuNoV GII.4 variants.

**Variants**	**Amino acid changes**	**SLAC**	**FEL**	**IFEL**
Farmington Hills 2002	Asn9His, Thr		°	
	Thr395Ala			°
	Ala395Thr			
Lanzou 2002	Gly255Ser			°
Asia 2003	Val413Ala			°
Hunter 2004	Arg340Gly		°	
Yerseke 2006a	Ser98Gly			°
Den Haag 2006b	Asn9Ser, Thr, His		°	°
	Pro357Asp			°
	Ser393Gly, Asn	°	°	°
	Gly393Ser			
	Asn412Asp, Ser	°	°	°
	Asp412Gly			
	His414Gln, Pro			°
	Pro414His			
Osaka 2007	Leu352Tyr, Phe			°
	Ser393Asn			°
	Asn407Gly, Ser			°
	Thr412Asp			°
Apeldoorn 2007	Ala359Thr, Ser		°	
New Orleans 2009	Pro294Ser, Thr	°	°	°
	Ser294Ala, Pro			
	Ala294Thr			
	Asn341Asp			°
	Asp341Asn			
	Glu376Asp, Val, Gln	°	°	°
	Asp376Val, Glu			
	Val376Asp, Glu, Ile			
	Ile413Thr, Val			°
	Thr413Ile			
Sydney 2012	Ile293Thr		°	
	Ser309Asn		°	°
	Asn309Ser			
	His373Arg, Asn		°	
	Ser393Gly, Asn, Thr	°	°	°
	Gly393Ser			
	Tyr460His		°	

*Positive selection sites were not found in the Camberwell 1994, US95_96, and Kaiso 2003 variants*.

**Table 5:** Number of negative selection sites in the present strains of HuNoV GII.4.

**Variants**	**Domains**^****^†^****^			**Total^^†^^**
	**Shell**	**P1**	**P2**	
Camberwell 1994	0	0	0	0
US95-96	4 (1.8%)	1 (0.5%)	1 (0.6%)	6 (1.1%)
Kaiso 2003	0	0	0	0
Farmington Hills 2002	0	0	0	0
Lanzou 2002	0	0	0	0
Asia 2003	0	0	0	0
Hunter 2004	0	0	0	0
Yerseke 2006a	0	0	0	0
Den Haag 2006b	34 (15.4%)	16 (9.0%)	9 (6.2%)	59 (10.9%)
Osaka 2007	4 (1.8%)	3 (1.6%)	4 (2.7%)	11 (2.03%)
Apeldoorn 2007	2 (0.9%)	0	0	2 (0.3%)
New Orleans 2009	10 (4.5%)	12 (6.7%)	3 (2.0%)	25 (4.6%)
Sydney 2012	3 (1.3%)	0	0	3 (0.5%)
All GII.4	140 (63.6%)	142 (80.2%)	87 (60.8%)	369 (68.3%)

† *The number of consensus sites by SLAC, FEL and IFEL methods is exhibited*.

Next, we mapped the conformational epitopes, positive selection, and amino acid substitutions on the VP1 structures in GII.4 variants. Most putative epitopes and substitutions were located on the exterior surface of the P2 domain in all variants. The epitopes overlapped with or were proximal to the substitution sites. Moreover, positive selection sites for shell and P1 domains existed at the exterior surface of the structure that was distal to the predicted epitopes. However, the positive selection sites for Den Haag 2006b, New Orleans 2009, and Sydney 2012 variants were located on the exterior surface of the epitopes (Figure 5). These results indicate that HuNoV GII.4 has evolved with frequent alteration of antigenicity. In addition, the circulation of GII.4 may also be associated with mechanisms other than immune escape.

In addition, we estimated the fluctuation in the size of infectious populations in HuNoV GII.4. The population size increased around 1994–1996 and 2005–2006. Moreover, Den Haag 2006b, New Orleans 2009, and Sydney 2012 variants accumulated infectious populations in 2005–2007, 2004–2010, and 2006–2011, respectively (Figure 6). These results suggest that GII.4 has a capacity to repeatedly produce highly epidemic variants. A previous report showed evidence of higher affinity to a broad range of HBGA types in the Den Haag 2006b strains compared with that in other variants (de Rougemont et al., 2011). Thus, the prevalence of HuNoV GII.4 may be associated with multiple factors, such as the capacity to bind to host receptors and immune evasion.

Overall, this study utilized limited numbers of GII.4 *VP1* sequences. For example, there were some biases among GII.4 variants in the amount of original data deposited in GenBank. Additionally, sequences with >98.9% identity were omitted. These biases may have affected the results of the bioinformatic analyses.

In conclusion, our findings show that the evolutionary pattern of GII.4 may be distinct among various variants. Moreover, our data may contribute to developing candidates of an efficient vaccine and prevalent prediction systems for HuNoV. In the near future, the accumulation of data from molecular epidemiological studies with continuous surveillance will be needed.

## Author contributions

TM, KK, and HK designed this study. TM, KN, YMa, NN, AR, TS, AY, MK, YMo, YS, and NS analyzed the data. TM, KN, YMa, and HK wrote the manuscript. KK and HK supervised this study. All authors read and approved the manuscript.

### Conflict of interest statement

The authors declare that the research was conducted in the absence of any commercial or financial relationships that could be construed as a potential conflict of interest.

## Footnotes

^1^http://www.ncbi.nlm.nih.gov/

^2^http://tree.bio.ed.ac.uk/software/tracer/
