# Supplementary material for: Molecular Evolution of the VP1 Gene in Human Norovirus GII.4 Variants in 1974–2015
Source: Front Microbiol. 2017 Dec 5;8:2399. doi: 10.3389/fmicb.2017.02399 (PMC5723339; doi:10.3389/fmicb.2017.02399)
Supplement: Supplementary file 1 [file DataSheet1.docx]

Supplementary Material

**Molecular evolution of the *VP1* gene in human norovirus GII.4** **variants in 1974**–**2015**

**Takumi Motoya, Koo Nagasawa, Yuki Matsushima, Noriko Nagata, Akihide Ryo, Tsuyoshi Sekizuka, Akifumi Yamashita, Makoto Kuroda, Yukio Morita, Yoshiyuki Suzuki, Nobuya Sasaki, Kazuhiko Katayama^*^, and Hirokazu Kimura^*^**

*** Correspondence:** Hirokazu Kimura: h-kimura@paz.ac.jp, Kazuhiko Katayama: katayama@lisci.kitasato-u.ac.jp

# Supplementary Figures


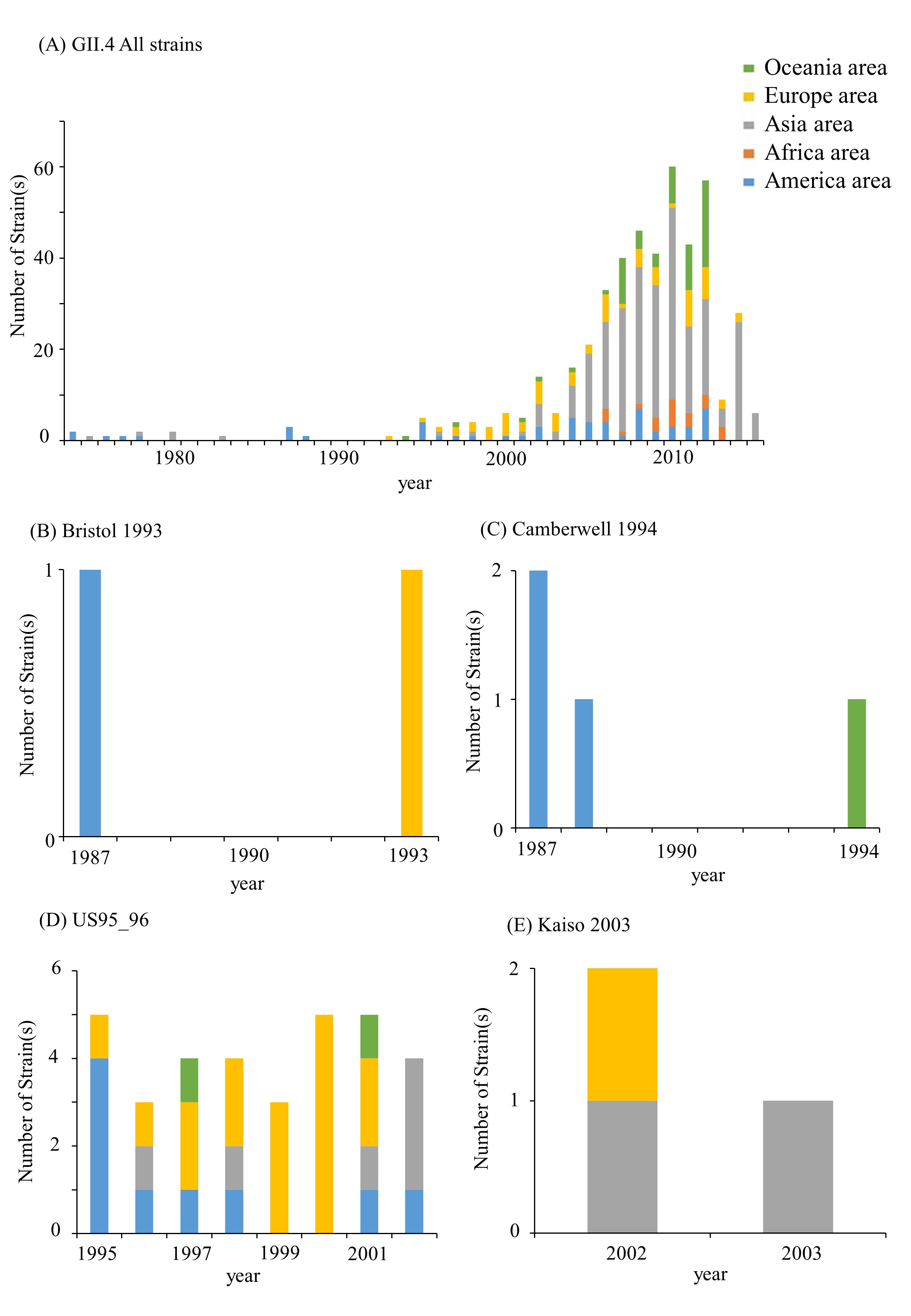


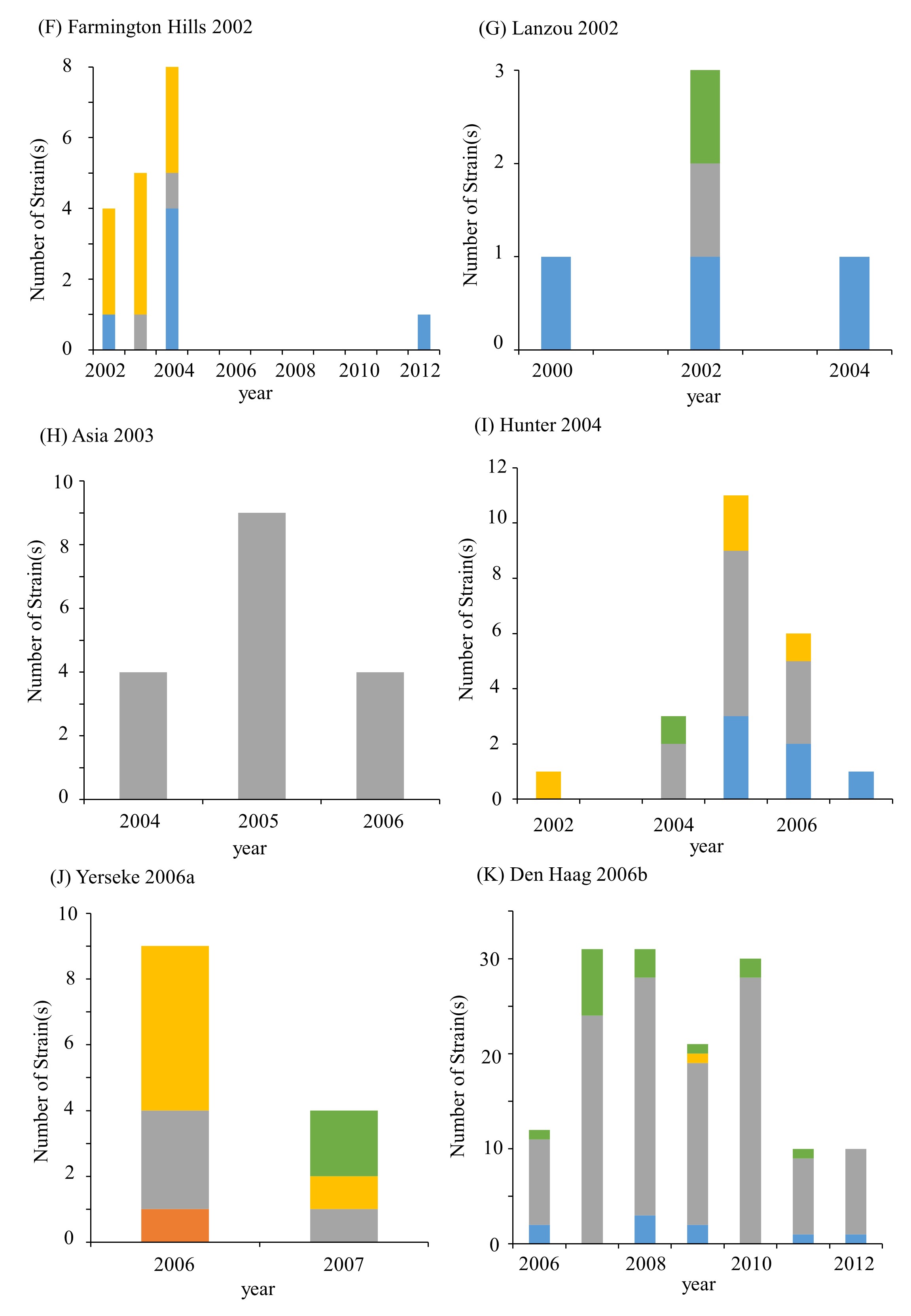


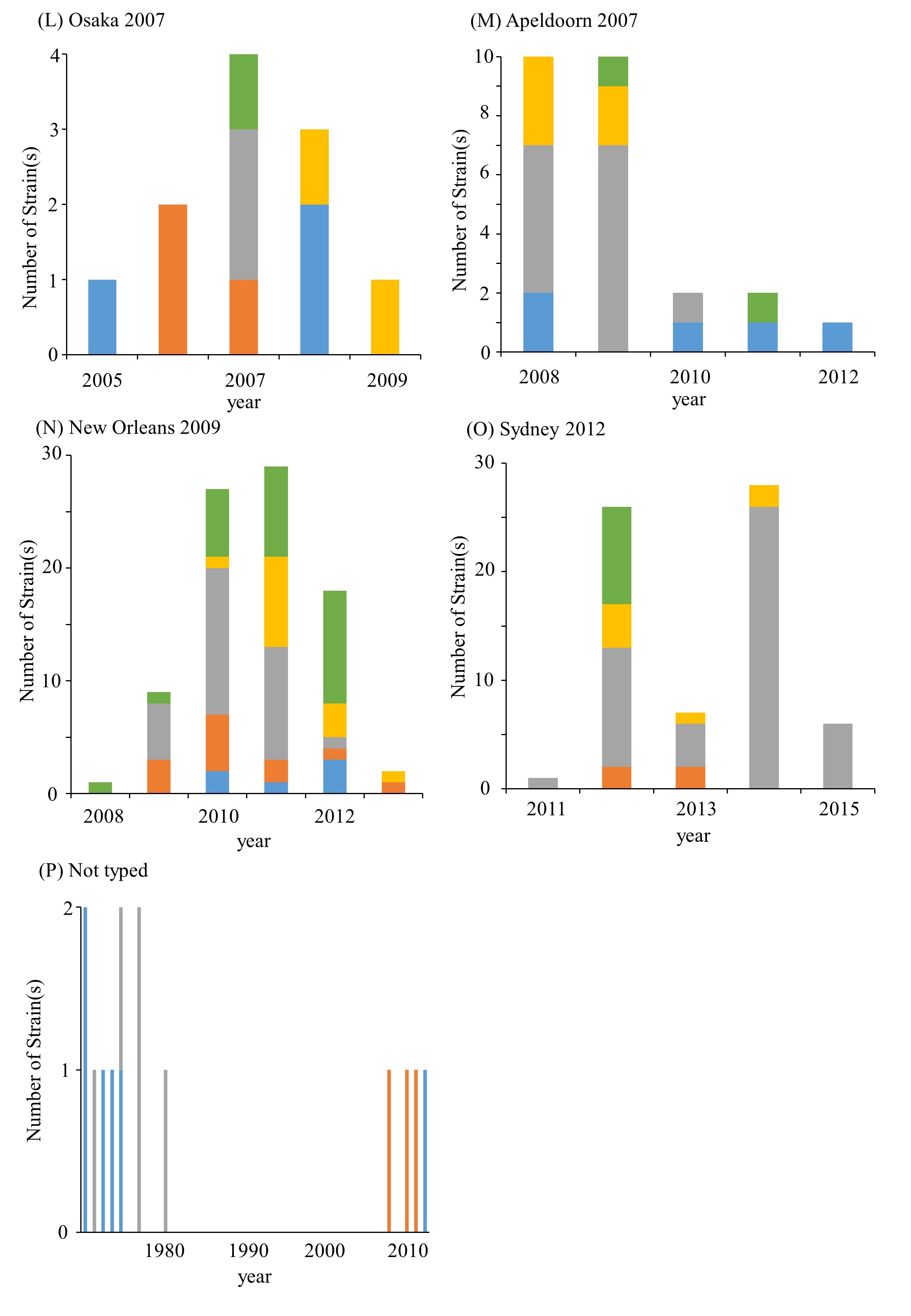


**Figure S1.** The collected information of HuNoV GII.4 strains used in this study. (A) GII.4 all strains; (B) Bristol 1993 variant strains; (C) Camberwell 1994 variant strains; (D) US95_96 variant strains; (E) Kaiso 2003 variant strains; (F) Farmington Hills 2002 variant strains; (G) Lanzou 2002 variant strains; (H) Asia 2003 variant strains; (I) Hunter 2004 variant strains; (J) Yerseke 2006a variant strains; (K) Den Haag 2006 variant strains; (L) Osaka 2007 variant strains; (M) Apeldoorn 2007 variant strains; (N) New Orleans 2009 variant strains; (O) Sydney 2012 variant strains; (P) Non-typed GII.4 strains. The y-axis represents the number of strain(s), while the x-axis denotes the collected years.


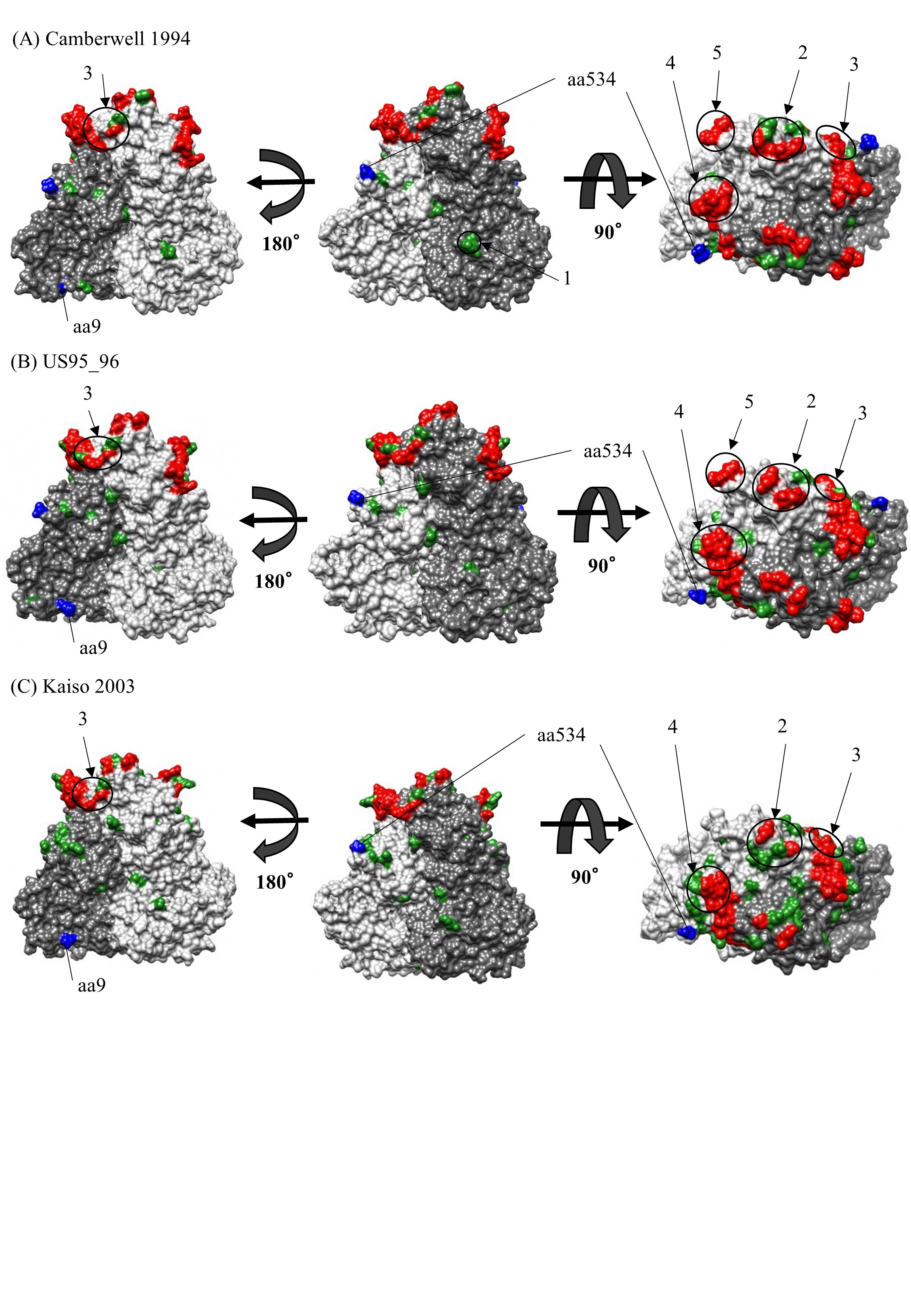


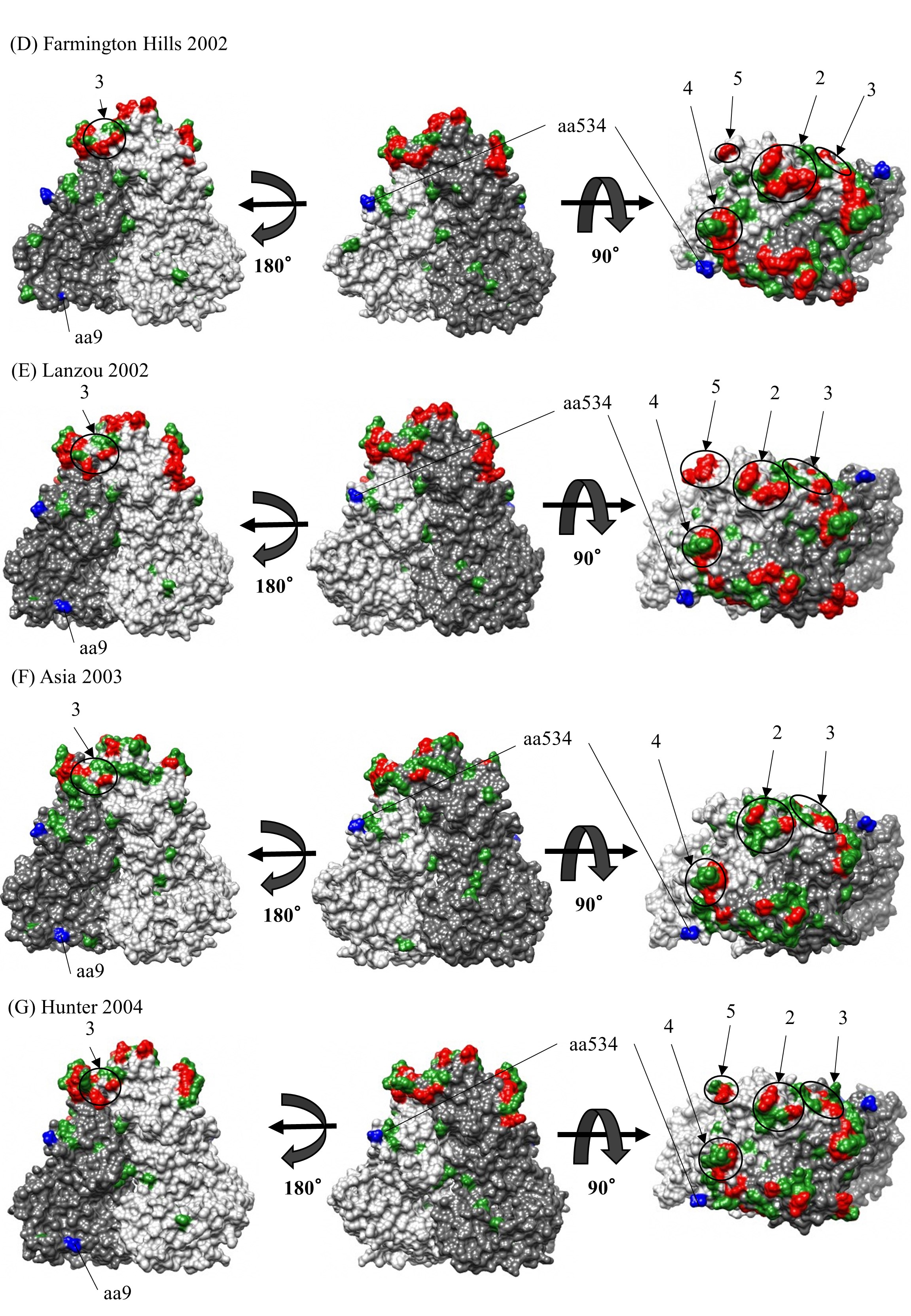

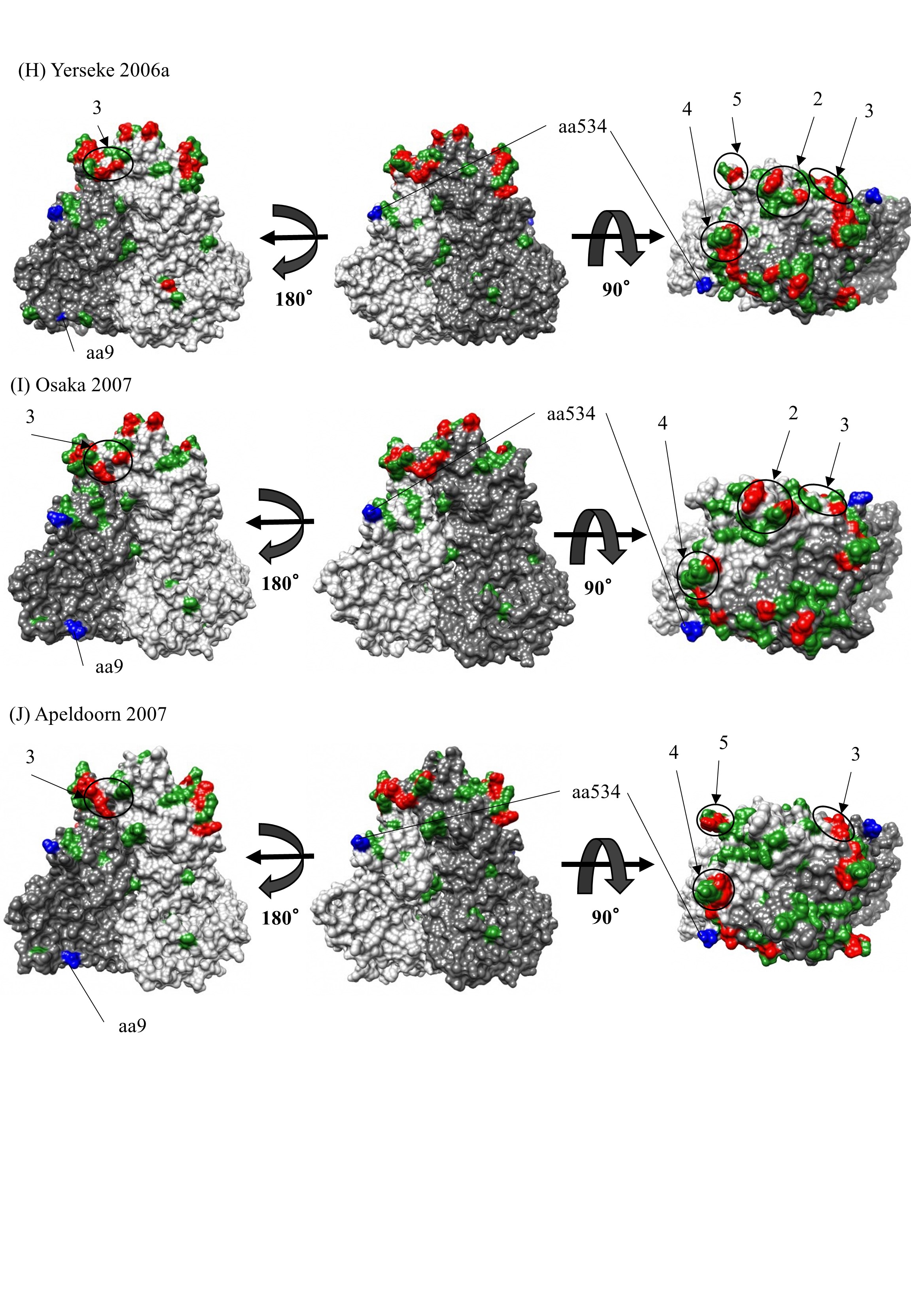


**Figure S2.** Structural models for the capsid VP1 protein of each HuNoV GII.4 variant. Three-dimensional VP1 dimer structures for the Camberwell 1994 (A), the US95_96 (B), the Kaiso 2003 (C), the Farmington Hills 2002 (D), the Lanzou 2002 (E), the Asia 2003 (F), the Hunter 2004 (G), the Yerseke 2006a (H), the Osaka 2007 (I) and the Apeldoorn 2007 (J) variants are shown. Chains that are composed of dimer structures are colored in gray (chain A) and dim gray (chain B). Predicted epitopes of each variant are colored in red and circled for regions. Positive selection sites are colored in blue for aa9 and aa534. Of note, aa6 could not be specified due to lack of structure modeling in N terminus. Amino acid substitutions of the other variants to a GII.4 Bristol 1993 strain are colored in green.

**Supplement Tables**

Table S1. Strains used in this study (Genotypes other than GII.4)

| GenBank  Accession No. | Genotypes | Names |
| --- | --- | --- |
| M87661 | GI.1 | Hu/GI.1/Norwalk/1968/US |
| U07611 | GII.1 | Hu/GII.1/Hawaii/1971/US |
| X81879 | GII.2 | Hu/GII.2/Melksham/1994/UK |
| U02030 | GII.3 | Hu/GII.3/Toronto24/1991/CA |
| AJ277607 | GII.5 | Hu/GII.5/Hillingdon/1990/UK |
| AB039778 | GII.6 | Hu/GII.6/Saitama/U16/1997/JP |
| AF414409 | GII.7 | Hu/GII.7/Gwynedd/273/1994/US |
| AB039780 | GII.8 | Hu/GII.8/Saitama/U25/1998/JP |
| AY038599 | GII.9 | Hu/GII.9/VA97207/1997/USA |
| AF427118 | GII.10 | Hu/GII.10/Erfurt/546/2000/DE |
| AB074893 | GII.11 | Sw/GII.11/Sw918/1997/JP |
| AB032758 | GII.12 | Hu/GII.12/Aichi/1996/JP |
| AY113106 | GII.13 | Hu/GII.13/Fayetteville/1998/US |
| AY130761 | GII.14 | Hu/GII.14/M7/1999/US |
| AY130762 | GII.15 | Hu/GII.15/J23/1999/US |
| AY502010 | GII.16 | Hu/GII.16/Tiffin/1999/USA |
| AY502009 | GII.17 | Hu/GII.17/CS-E1/2002/USA |
| AY823304 | GII.18 | Sw/GII.18/OH-QW101/2003/US |
| AY823306 | GII.19 | Sw/GII.19/OH-QW170/2003/US |
| AB542917 | GII.20 | Hu/GII.20/OC07118/2007/JP |
| AB542915 | GII.21 | Hu/GII.21/OC05024/2005/JP |
| AB083780 | GII.22 | Hu/GII.22/YURI/2002/JP |

Table S1 (continued). Strains used in this study (GII.4 genotype)

| GenBank  Accession No. | | GII.4 variants | | Names | |
| --- | --- | --- | --- | --- | --- |
| FJ537137 | | Bristol1993 | | Hu/GII.4/Bristol1993/CHDC4108/1987/US | |
| X76716 | | Bristol1993 | | Hu/GII.4/Bristol1993/Bristol/1993/UK | |
| AF145896 | | Camberwell1994 | | Hu/GII.4/Camberwell1994/101922/1994/AUS | |
| AY030098 | | Camberwell1994 | | Hu/GII.4/Camberwell1994/MD134-7/1987/US | |
| FJ537136 | | Camberwell1994 | | Hu/GII.4/Camberwell1994/CHDC3967/1988/US | |
| JX289821 | | Camberwell1994 | | Hu/GII.4/Camberwell1994/MD120-12/1987/USA | |
| AB078336 | | US95-96 | | Hu/GII.4/US95-96/Narita104/2002/JP | |
| AB083781 | | US95-96 | | Hu/GII.4/US95-96/YURI/Akita/32073/2002/JP | |
| AB294778 | | US95-96 | | Hu/GII.4/US95-96/Matsudo/021071/2002/JP | |
| AB303922 | | US95-96 | | Hu/GII.4/US95-96/Tiel001/1995/NL | |
| AB303924 | | US95-96 | | Hu/GII.4/US95-96/Waddinxveen016/2000/NL | |
| AB303925 | | US95-96 | | Hu/GII.4/US95-96/Leeuwarden043/2000/NL | |
| AB303926 | | US95-96 | | Hu/GII.4/US95-96/Schiedam018/2001/NL | |
| AB504306 | | US95-96 | | Hu/GII.4/US95-96/Hiroshima/19/2001/JPN | |
| AF080552 | | US95-96 | | Hu/GII.4/US95-96/358/96015107/1996/FL | |
| AF080554 | | US95-96 | | Hu/GII.4/US95-96/366/96019554/1996/ID | |
| AF406793 | | US95-96 | | Hu/GII.4/US95-96/DOUG4770/2001/AUS | |
| AF414424 | | US95-96 | | Hu/GII.4/US95-96/Miami_Beach/326/1995/US | |
| AF414425 | | US95-96 | | Hu/GII.4/US95-96/Burwash_Landing/331/1995/US | |
| AF425763 | | US95-96 | | Hu/GII.4/US95-96/Berlin/159/1998/DE | |
| AF425764 | | US95-96 | | Hu/GII.4/US95-96/Berlin/238/1998/DE | |
| AF425766 | | US95-96 | | Hu/GII.4/US95-96/Dillingen_259/2001/DE | |
| AF427113 | | US95-96 | | Hu/GII.4/US95-96/Oberschleissheim/112/1999/DE | |
| AF427114 | | US95-96 | | Hu/GII.4/US95-96/Frankfurt/170/1999/DE | |
| AF427115 | | US95-96 | | Hu/GII.4/US95-96/Ludwigslust/218/1999/DE | |
| AF427120 | | US95-96 | | Hu/GII.4/US95-96/Beeskow/124/2000/DE | |
| AF427121 | | US95-96 | | Hu/GII.4/US95-96/Koenigswusterhausen/130/2000/DE | |
| AF427122 | | US95-96 | | Hu/GII.4/US95-96/Berlin/491/2000/DE | |
| AF472623 | | US95-96 | | Hu/GII.4/US95-96/DIJON171/1996/FR | |
| AJ277619 | | US95-96 | | Hu/GII.4/US95-96/Symgreen/1995/UK | |
| AY038600 | | US95-96 | | Hu/GII.4/US95-96/VA98387/1998/USA | |
| AY081134 | | US95-96 | | Hu/GII.4/US95-96/Mora/1997/SE | |
| AY741811 | | US95-96 | | Hu/GII.4/US95-96/Dresden174/1997/GE | |
| DQ078829 | | US95-96 | | Hu/GII.4/US95-96/Sydney348/1997/AU | |
| DQ975270 | | US95-96 | | Hu/GII.4/US95-96/Osaka/1998/JPN | |
| EU078410 | | US95-96 | | Hu/GII.4/US95-96/GCanyon/2002/USA | |
| EU105469 | | US95-96 | | Hu/GII.4/US95-96/HS66/2001/US | |
| FJ411169 | | US95-96 | | Hu/GII.4/US95-96/Wellington/1995/USA | |
| JQ478407 | | US95-96 | | Hu/GII.4/US95-96/1997/USA | |
| AB186065 | | Kaiso2003 | | Hu/GII.4/Kaiso2003/Osaka/OC02202/2002/JP | |
| AB294779 | | Kaiso2003 | | Hu/GII.4/Kaiso2003/Chiba/030556/2003/JP | |
| AB303929 | | Kaiso2003 | | Hu/GII.4/Kaiso2003/EmmenE006/2002/NL | |
| AB294780 | | FarmingtonHills2002 | | Hu/GII.4/FarmingtonHills2002/Chiba/040095/2003/JP | |
| AB303928 | | FarmingtonHills2002 | | Hu/GII.4/FarmingtonHills2002/WeertE022/2002/NL | |
| AB303930 | | FarmingtonHills2002 | | Hu/GII.4/FarmingtonHills2002/Heerlen003/2003/NL | |
| AB303931 | | FarmingtonHills2002 | | Hu/GII.4/FarmingtonHills2002/Apeldoorn023/2003/NL | |
| AB303932 | | FarmingtonHills2002 | | Hu/GII.4/FarmingtonHills2002/DenHaag001/2003/NL | |
| AB303934 | | FarmingtonHills2002 | | Hu/GII.4/FarmingtonHills2002/DenHelder003/2004/NL | |
| AB303935 | | FarmingtonHills2002 | | Hu/GII.4/FarmingtonHills2002/Middelburg007/2004/NL | |
| AB303936 | | FarmingtonHills2002 | | Hu/GII.4/FarmingtonHills2002/Elsloo012/2004/NL | |
| AY502019 | | FarmingtonHills2002 | | Hu/GII.4/FarmingtonHills2002/Anchorage/2002/USA | |
| AY588018 | | FarmingtonHills2002 | | Hu/GII.4/FarmingtonHills2002/Oxford/B5S13/2002/UK | |
| AY588021 | | FarmingtonHills2002 | | Hu/GII.4/FarmingtonHills2002/Oxford/B6S6/2003/UK | |
| EU078413 | | FarmingtonHills2002 | | Hu/GII.4/FarmingtonHills2002/GA04/2004/USA | |
| FJ538900 | | FarmingtonHills2002 | | Hu/GII.4/FarmingtonHills2002/Dijon/E872/2002/FRA | |
| JQ478408 | | FarmingtonHills2002 | | Hu/GII.4/FarmingtonHills2002/Farmington_Hills/2004/USA | |
| JQ798158 | | FarmingtonHills2002 | | Hu/GII.4/FarmingtonHills2002/5M/2004/USA | |
| JX126912 | | FarmingtonHills2002 | | Hu/GII.4/FarmingtonHills2002/Ohio/7I/2012/USA | |
| JX445152 | | FarmingtonHills2002 | | Hu/GII.4/FarmingtonHills2002/AlbertaEI131/2004/CA | |
| JX459596 | FarmingtonHills2002 | | Hu/GII.4/FarmingtonHills2002/SG4016-09/2004/SG | |  |

Table S1 (continued). Strains used in this study (GII.4 genotype)

| GenBank  Accession No. | GII.4 variants | Names |
| --- | --- | --- |
| DQ078820 | Lanzou2002 | Hu/GII.4/Lanzou2002/Sydney917J/2002/AU |
| DQ364459 | Lanzou2002 | Hu/GII.4/Lanzou2002/35666/2002/China |
| EU078412 | Lanzou2002 | Hu/GII.4/Lanzou2002/FL04/2004/USA |
| EU310927 | Lanzou2002 | Hu/GII.4/Lanzou2002/Houston/TCH186/2002/US |
| FJ411170 | Lanzou2002 | Hu/GII.4/Lanzou2002/Henry/2000/USA |
| AB220921 | Asia2003 | Hu/GII.4/Asia2003/Chiba/2005/JP |
| AB220922 | Asia2003 | Hu/GII.4/Asia2003/Sakai/2005/JP |
| AB220923 | Asia2003 | Hu/GII.4/Asia2003/Ehime/2005/JP |
| AB294782 | Asia2003 | Hu/GII.4/Asia2003/Chiba/2004/JP |
| AB294787 | Asia2003 | Hu/GII.4/Asia2003/Ichikawa/2005/JP |
| AB294791 | Asia2003 | Hu/GII.4/Asia2003/Inba/2006/JP |
| AB447448 | Asia2003 | Hu/GII.4/Asia2003/Sakai2/2006/JP |
| AB504307 | Asia2003 | Hu/GII.4/Asia2003/Hiroshima/2004/JPN |
| AB504313 | Asia2003 | Hu/GII.4/Asia2003/Hiroshima/2005/JPN |
| AB504314 | Asia2003 | Hu/GII.4/Asia2003/Hiroshima/2005/JPN |
| DQ369797 | Asia2003 | Hu/GII.4/Asia2003/Guangzhou/NVgz01/2006/CHN |
| EF535854 | Asia2003 | Hu/GII.4/Asia2003/Guangzhou/2006/China |
| EU839581 | Asia2003 | Hu/GII.4/Asia2003/Beijing/2004/CHN |
| EU839586 | Asia2003 | Hu/GII.4/Asia2003/Beijing/2005/CHN |
| EU839587 | Asia2003 | Hu/GII.4/Asia2003/Beijing/2005/CHN |
| HM802548 | Asia2003 | Hu/GII.4/Asia2003/Hong_Kong/2005/CHN |
| HM802551 | Asia2003 | Hu/GII.4/Asia2003/Hong_Kong/2004/CHN |
| AB294785 | Hunter2004 | Hu/GII.4/Hunter2004/Inba/050590/2005/JP |
| AB294788 | Hunter2004 | Hu/GII.4/Hunter2004/Sanbu/050878/2006/JP |
| AB385629 | Hunter2004 | Hu/GII.4/Hunter2004/RotterdamP2D182/2005/NL |
| AB385635 | Hunter2004 | Hu/GII.4/Hunter2004/RotterdamP5D36/2005/NL |
| DQ078794 | Hunter2004 | Hu/GII.4/Hunter2004/Sydney284E/2004/AU |
| EF126962 | Hunter2004 | Hu/GII.4/Hunter2004/DenHaag54/2006/NL |
| EF202568 | Hunter2004 | Hu/GII.4/Hunter2004/Toronto/SK/2005/CAN |
| EU078415 | Hunter2004 | Hu/GII.4/Hunter2004/Ryndam/2005/USA |
| EU839584 | Hunter2004 | Hu/GII.4/Hunter2004/Beijing/221/2005/CHN |
| EU876890 | Hunter2004 | Hu/GII.4/Hunter2004/Dijon-E1057/2002/FRA |
| EU916956 | Hunter2004 | Hu/GII.4/Hunter2004/Monastir_8655/2007/TUN |
| EU921338 | Hunter2004 | Hu/GII.4/Hunter2004/Pune-PC09/2005/IN |
| EU921344 | Hunter2004 | Hu/GII.4/Hunter2004/Pune/PC15/2006/India |
| HM802543 | Hunter2004 | Hu/GII.4/Hunter2004/Hong_Kong/CU050106/2005/CHN |
| HM802544 | Hunter2004 | Hu/GII.4/Hunter2004/Hong_Kong/CU051146/2005/CHN |
| HQ456320 | Hunter2004 | Hu/GII.4/Hunter2004/04R-2/2004/TW |
| JF827296 | Hunter2004 | Hu/GII.4/Hunter2004/Houston/TCH492/2005/US |
| JN400599 | Hunter2004 | Hu/GII.4/Hunter2004/CGMH01/2006/TW |
| JX445153 | Hunter2004 | Hu/GII.4/Hunter2004/AlbertaEI142/2006/CA |
| JX459595 | Hunter2004 | Hu/GII.4/Hunter2004/SG4013-09/2004/SG |
| JX459597 | Hunter2004 | Hu/GII.4/Hunter2004/SG4033-01/2005/SG |
| KC715794 | Hunter2004 | Hu/GII.4/Hunter2004/DF-01/2006/Brazil |
| AB385632 | Yerseke2006a | Hu/GII.4/Yerseke2006a/RotterdamP4D0/2006/NL |
| AB385638 | Yerseke2006a | Hu/GII.4/Yerseke2006a/RotterdamP6D33/2006/NL |
| AB385640 | Yerseke2006a | Hu/GII.4/Yerseke2006a/RotterdamP7D119/2007/NL |
| AB447458 | Yerseke2006a | Hu/GII.4/Yerseke2006a/Saga5/2006/JP |
| AB541267 | Yerseke2006a | Hu/GII.4/Yerseke2006a/Hokkaido5/2007/JP |
| EF126963 | Yerseke2006a | Hu/GII.4/Yerseke2006a/Yerseke38/2006/NL |
| EF187497 | Yerseke2006a | Hu/GII.4/Yerseke2006a/Kenepuru/NZ327/2006/NZL |
| EU876886 | Yerseke2006a | Hu/GII.4/Yerseke2006a/Cairo6/2006/EGY |
| EU876894 | Yerseke2006a | Hu/GII.4/Yerseke2006a/Dijon-E1501/2006/FRA |
| JQ613511 | Yerseke2006a | Hu/GII.4/Yerseke2006a/WA223N/2007/AU |
| JQ613517 | Yerseke2006a | Hu/GII.4/Yerseke2006a/WA210Z/2007/AU |
| JX459607 | Yerseke2006a | Hu/GII.4/Yerseke2006a/SG4061-10/2006/SG |
| JX459612 | Yerseke2006a | Hu/GII.4/Yerseke2006a/SG4058-12/2006/SG |
| AB291542 | DenHaag2006b | Hu/GII.4/DenHaag2006b/Kobe034/2006/JP |

Table S1 (continued). Strains used in this study (GII.4 genotype)

| GenBank  Accession No. | GII.4 variants | Names |
| --- | --- | --- |
| AB294794 | DenHaag2006b | Hu/GII.4/DenHaag2006b/Narashino/2006/JP |
| AB447449 | DenHaag2006b | Hu/GII.4/DenHaag2006b/Sakai3/2006/JP |
| AB447454 | DenHaag2006b | Hu/GII.4/DenHaag2006b/Ehime2/2006/JP |
| AB504323 | DenHaag2006b | Hu/GII.4/DenHaag2006b/Hiroshima/2007/JPN |
| AB541204 | DenHaag2006b | Hu/GII.4/DenHaag2006b/Aichi2/2008/JP |
| AB541212 | DenHaag2006b | Hu/GII.4/DenHaag2006b/Akita1/2008/JP |
| AB541215 | DenHaag2006b | Hu/GII.4/DenHaag2006b/Akita3/2008/JP |
| AB541219 | DenHaag2006b | Hu/GII.4/DenHaag2006b/Aomori1/2008/JP |
| AB541225 | DenHaag2006b | Hu/GII.4/DenHaag2006b/Aomori4/2008/JP |
| AB541227 | DenHaag2006b | Hu/GII.4/DenHaag2006b/Aomori5/2008/JP |
| AB541231 | DenHaag2006b | Hu/GII.4/DenHaag2006b/Chiba2/2008/JP |
| AB541234 | DenHaag2006b | Hu/GII.4/DenHaag2006b/Chiba5/2007/JP |
| AB541240 | DenHaag2006b | Hu/GII.4/DenHaag2006b/Ehime3/2008/JP |
| AB541241 | DenHaag2006b | Hu/GII.4/DenHaag2006b/Ehime4/2007/JP |
| AB541244 | DenHaag2006b | Hu/GII.4/DenHaag2006b/Fukui1/2008/JP |
| AB541248 | DenHaag2006b | Hu/GII.4/DenHaag2006b/Fukui4/2008/JP |
| AB541250 | DenHaag2006b | Hu/GII.4/DenHaag2006b/Fukui5/2008/JP |
| AB541253 | DenHaag2006b | Hu/GII.4/DenHaag2006b/Hiroshima2/2007/JP |
| AB541254 | DenHaag2006b | Hu/GII.4/DenHaag2006b/Hiroshima2/2008/JP |
| AB541260 | DenHaag2006b | Hu/GII.4/DenHaag2006b/Hokkaido1/2007/JP |
| AB541262 | DenHaag2006b | Hu/GII.4/DenHaag2006b/Hokkaido2/2007/JP |
| AB541263 | DenHaag2006b | Hu/GII.4/DenHaag2006b/Hokkaido2/2008/JP |
| AB541264 | DenHaag2006b | Hu/GII.4/DenHaag2006b/Hokkaido3/2008/JP |
| AB541265 | DenHaag2006b | Hu/GII.4/DenHaag2006b/Hokkaido4/2007/JP |
| AB541271 | DenHaag2006b | Hu/GII.4/DenHaag2006b/Iwate3/2007/JP |
| AB541290 | DenHaag2006b | Hu/GII.4/DenHaag2006b/Miyazaki2/2007/JP |
| AB541306 | DenHaag2006b | Hu/GII.4/DenHaag2006b/Nagano3/2008/JP |
| AB541308 | DenHaag2006b | Hu/GII.4/DenHaag2006b/Nagano5/2007/JP |
| AB541314 | DenHaag2006b | Hu/GII.4/DenHaag2006b/Niigata3/2008/JP |
| AB541315 | DenHaag2006b | Hu/GII.4/DenHaag2006b/Niigata4/2007/JP |
| AB541337 | DenHaag2006b | Hu/GII.4/DenHaag2006b/Saga5/2007/JP |
| AB541339 | DenHaag2006b | Hu/GII.4/DenHaag2006b/Sakai1/2007/JP |
| AB541340 | DenHaag2006b | Hu/GII.4/DenHaag2006b/Sakai1/2008/JP |
| AB541345 | DenHaag2006b | Hu/GII.4/DenHaag2006b/Sakai4/2008/JP |
| AB541348 | DenHaag2006b | Hu/GII.4/DenHaag2006b/Shimane2/2008/JP |
| AB541355 | DenHaag2006b | Hu/GII.4/DenHaag2006b/Toyama2/2007/JP |
| AB541358 | DenHaag2006b | Hu/GII.4/DenHaag2006b/Toyama3/2008/JP |
| AB629942 | DenHaag2006b | Hu/GII.4/DenHaag2006b/Tokyo/2010/JPN |
| AB663685 | DenHaag2006b | Hu/GII.4/DenHaag2006b/Wakayama/T162/2010/JP |
| AB663686 | DenHaag2006b | Hu/GII.4/DenHaag2006b/Wakayama/T164/2010/JP |
| AB933646 | DenHaag2006b | Hu/GII.4/DenHaag2006b/Shimane1/2009/JP |
| AB933664 | DenHaag2006b | Hu/GII.4/DenHaag2006b/Nagano1/2010/JP |
| AB933666 | DenHaag2006b | Hu/GII.4/DenHaag2006b/Osaka2/2010/JP |
| AB933668 | DenHaag2006b | Hu/GII.4/DenHaag2006b/Osaka4/2010/JP |
| AB933672 | DenHaag2006b | Hu/GII.4/DenHaag2006b/Hiroshima4/2010/JP |
| AB933675 | DenHaag2006b | Hu/GII.4/DenHaag2006b/Hokkaido4/2010/JP |
| AB933682 | DenHaag2006b | Hu/GII.4/DenHaag2006b/Nagano3/2010/JP |
| AB933685 | DenHaag2006b | Hu/GII.4/DenHaag2006b/Ehime3/2010/JP |
| AB933690 | DenHaag2006b | Hu/GII.4/DenHaag2006b/Miyazaki4/2010/JP |
| AB933698 | DenHaag2006b | Hu/GII.4/DenHaag2006b/Hiroshimacity2/2011/JP |
| AB933705 | DenHaag2006b | Hu/GII.4/DenHaag2006b/Ehime2/2011/JP |
| AB933717 | DenHaag2006b | Hu/GII.4/DenHaag2006b/Toyama5/2011/JP |
| AB933727 | DenHaag2006b | Hu/GII.4/DenHaag2006b/Chiba10/2011/JP |
| EF684915 | DenHaag2006b | Hu/GII.4/DenHaag2006b/Shellharbour/NSW696T/2006/AUS |
| EU078418 | DenHaag2006b | Hu/GII.4/DenHaag2006b/Mississip/2006/USA |
| EU078420 | DenHaag2006b | Hu/GII.4/DenHaag2006b/MT01/2006/USA |
| EU839588 | DenHaag2006b | Hu/GII.4/DenHaag2006b/Beijing/2006/CHN |
| EU839589 | DenHaag2006b | Hu/GII.4/DenHaag2006b/Beijing/2006/CHN |
| EU839593 | DenHaag2006b | Hu/GII.4/DenHaag2006b/Beijing/2007/CHN |
| EU921386 | DenHaag2006b | Hu/GII.4/DenHaag2006b/Pune-PC49/2007/IN |

Table S1 (continued). Strains used in this study (GII.4 genotype)

| GenBank  Accession No. | GII.4 variants | Names |
| --- | --- | --- |
| GQ246799 | DenHaag2006b | Hu/GII.4/DenHaag2006b/Dijon/2009/FRA |
| GQ845024 | DenHaag2006b | Hu/GII.4/DenHaag2006b/Rathmines/NSW287R/2007/AUS |
| GQ845322 | DenHaag2006b | Hu/GII.4/DenHaag2006b/VIC3863/2007/AU |
| GQ845329 | DenHaag2006b | Hu/GII.4/DenHaag2006b/NSW523R/2007/AU |
| GQ845341 | DenHaag2006b | Hu/GII.4/DenHaag2006b/NSW587V/2007/AU |
| GQ845366 | DenHaag2006b | Hu/GII.4/DenHaag2006b/Westmead/NSW3639/2008/AUS |
| GQ856448 | DenHaag2006b | Hu/GII.4/DenHaag2006b/Beijing/2007/CHN |
| GQ856449 | DenHaag2006b | Hu/GII.4/DenHaag2006b/Beijing/2007/CHN |
| GQ856451 | DenHaag2006b | Hu/GII.4/DenHaag2006b/Beijing/2007/CHN |
| GQ856452 | DenHaag2006b | Hu/GII.4/DenHaag2006b/Beijing/2007/CHN |
| GQ856455 | DenHaag2006b | Hu/GII.4/DenHaag2006b/Beijing/2007/CHN |
| GQ856456 | DenHaag2006b | Hu/GII.4/DenHaag2006b/Beijing/2007/CHN |
| GQ856457 | DenHaag2006b | Hu/GII.4/DenHaag2006b/Beijing/2008/CHN |
| GU325839 | DenHaag2006b | Hu/GII.4/DenHaag2006b/HS194/2009/US |
| GU390900 | DenHaag2006b | Hu/GII.4/DenHaag2006b/Chungnam/2008/Kor |
| HQ456332 | DenHaag2006b | Hu/GII.4/DenHaag2006b/08-E/2006/TW |
| HQ456344 | DenHaag2006b | Hu/GII.4/DenHaag2006b/09-N-1/2009/TW |
| JN400603 | DenHaag2006b | Hu/GII.4/DenHaag2006b/CGMH05/2006/TW |
| JN400613 | DenHaag2006b | Hu/GII.4/DenHaag2006b/CGMH15/2007/TW |
| JN400617 | DenHaag2006b | Hu/GII.4/DenHaag2006b/CGMH19/2009/TW |
| JQ613508 | DenHaag2006b | Hu/GII.4/DenHaag2006b/NSW721I/2007/AU |
| JQ613510 | DenHaag2006b | Hu/GII.4/DenHaag2006b/NSW833M/2007/AU |
| JQ613512 | DenHaag2006b | Hu/GII.4/DenHaag2006b/WA145Y/2007/AU |
| JQ613519 | DenHaag2006b | Hu/GII.4/DenHaag2006b/NSW3912/2008/AU |
| JQ613522 | DenHaag2006b | Hu/GII.4/DenHaag2006b/NSW764Q/2008/AU |
| JQ613525 | DenHaag2006b | Hu/GII.4/DenHaag2006b/NSW8190/2009/AU |
| JQ613562 | DenHaag2006b | Hu/GII.4/DenHaag2006b/NSW827D/2010/AU |
| JQ613572 | DenHaag2006b | Hu/GII.4/DenHaag2006b/StVincents/NSW217I/2010/AU |
| JX155737 | DenHaag2006b | Hu/GII.4/DenHaag2006b/Xian/P19/2010/CHN |
| JX155739 | DenHaag2006b | Hu/GII.4/DenHaag2006b/Xian/P49/2010/CHN |
| JX155742 | DenHaag2006b | Hu/GII.4/DenHaag2006b/Xian/P143/2010/CHN |
| JX155743 | DenHaag2006b | Hu/GII.4/DenHaag2006b/Xian/P152/2010/CHN |
| JX155745 | DenHaag2006b | Hu/GII.4/DenHaag2006b/Xian/P158/2010/CHN |
| JX155746 | DenHaag2006b | Hu/GII.4/DenHaag2006b/Xian/P185/2010/CHN |
| JX155747 | DenHaag2006b | Hu/GII.4/DenHaag2006b/Xian/C6/2010/CHN |
| JX155748 | DenHaag2006b | Hu/GII.4/DenHaag2006b/Xian/C12/2010/CHN |
| JX155750 | DenHaag2006b | Hu/GII.4/DenHaag2006b/Xian/C21/2010/CHN |
| JX155752 | DenHaag2006b | Hu/GII.4/DenHaag2006b/Xian/C31/2010/CHN |
| JX439841 | DenHaag2006b | Hu/GII.4/DenHaag2006b/Seoul1321/2010/KOR |
| JX439842 | DenHaag2006b | Hu/GII.4/DenHaag2006b/Seoul1351/2010/KOR |
| JX439843 | DenHaag2006b | Hu/GII.4/DenHaag2006b/Seoul1431/2010/KOR |
| JX445160 | DenHaag2006b | Hu/GII.4/DenHaag2006b/AlbertaEI102/2008/CA |
| JX445162 | DenHaag2006b | Hu/GII.4/DenHaag2006b/AlbertaEI425/2008/CA |
| JX445163 | DenHaag2006b | Hu/GII.4/DenHaag2006b/AlbertaEI109/2009/CA |
| JX459603 | DenHaag2006b | Hu/GII.4/DenHaag2006b/SG4019-09/2006/SG |
| JX459622 | DenHaag2006b | Hu/GII.4/DenHaag2006b/SG4052-08/2007/SG |
| JX459629 | DenHaag2006b | Hu/GII.4/DenHaag2006b/SG4069-01/2008/SG |
| JX459632 | DenHaag2006b | Hu/GII.4/DenHaag2006b/SG4092-11/2008/SG |
| JX459639 | DenHaag2006b | Hu/GII.4/DenHaag2006b/SG4028-07/2009/SG |
| JX459648 | DenHaag2006b | Hu/GII.4/DenHaag2006b/SG4058-10/2009/SG |
| JX459905 | DenHaag2006b | Hu/GII.4/DenHaag2006b/Randwick/NSW938K/2011/AU |
| JX984946 | DenHaag2006b | Hu/GII.4/DenHaag2006b/GZ2010-L26/Guangzhou/2010/CHN |
| JX984947 | DenHaag2006b | Hu/GII.4/DenHaag2006b/GZ2010-L32/Guangzhou/2010/CHN |
| JX984951 | DenHaag2006b | Hu/GII.4/DenHaag2006b/GZ2010-L88/Guangzhou/2011/CHN |
| KC175342 | DenHaag2006b | Hu/GII.4/DenHaag2006b/Norwalk/10034/2009/VNM |
| KC175357 | DenHaag2006b | Hu/GII.4/DenHaag2006b/Norwalk/10148/2009/VNM |
| KC175372 | DenHaag2006b | Hu/GII.4/DenHaag2006b/Norwalk/10204/2009/VNM |
| KC175374 | DenHaag2006b | Hu/GII.4/DenHaag2006b/Norwalk/10223/2009/VNM |

Table S1 (continued). Strains used in this study (GII.4 genotype)

| GenBank  Accession No. | GII.4 variants | Names |
| --- | --- | --- |
| KC175378 | DenHaag2006b | Hu/GII.4/DenHaag2006b/Norwalk/10247/2009/VNM |
| KC409255 | DenHaag2006b | Hu/GII.4/DenHaag2006b/Ho_Chi_Minh/20154/2009/VNM |
| KC409271 | DenHaag2006b | Hu/GII.4/DenHaag2006b/Ho_Chi_Minh/20190/2009/VNM |
| KC409278 | DenHaag2006b | Hu/GII.4/DenHaag2006b/Ho_Chi_Minh/20208/2009/VNM |
| KC409289 | DenHaag2006b | Hu/GII.4/DenHaag2006b/Ho_Chi_Minh/20302/2009/VNM |
| KC409298 | DenHaag2006b | Hu/GII.4/DenHaag2006b/Ho_Chi_Minh/20448/2010/VNM |
| KC517360 | DenHaag2006b | Hu/GII.4/DenHaag2006b/New_Taipei/CGMH50/2011/TW |
| KC517366 | DenHaag2006b | Hu/GII.4/DenHaag2006b/New_Taipei/CGMH56/2012/TW |
| KC517368 | DenHaag2006b | Hu/GII.4/DenHaag2006b/New_Taipei/CGMH58/2012/TW |
| KC517372 | DenHaag2006b | Hu/GII.4/DenHaag2006b/New_Taipei/CGMH62/2012/TW |
| KC517373 | DenHaag2006b | Hu/GII.4/DenHaag2006b/Taoyuan/CGMH63/2012/TW |
| KC576912 | DenHaag2006b | Hu/GII.4/DenHaag2006b/NIHIC4.1/2011/USA |
| KC990829 | DenHaag2006b | Hu/GII.4/DenHaag2006b/092895/2008/USA |
| KF429777 | DenHaag2006b | Hu/GII.4/DenHaag2006b/NIHIC27.1/2012/USA |
| KJ196287 | DenHaag2006b | Hu/GII.4/DenHaag2006b/Shimada/ASC96/2010/JP |
| KJ541743 | DenHaag2006b | Hu/GII.4/DenHaag2006b/SGU-110421/2011/KOR |
| KM198485 | DenHaag2006b | Hu/GII.4/DenHaag2006b/10148/2009/VNM |
| KM198533 | DenHaag2006b | Hu/GII.4/DenHaag2006b/30241/2009/VNM |
| KM198539 | DenHaag2006b | Hu/GII.4/DenHaag2006b/C2H-36/2011/VNM |
| KM198570 | DenHaag2006b | Hu/GII.4/DenHaag2006b/20146/2009/VNM |
| KT033904 | DenHaag2006b | Hu/GII.4/DenHaag2006b/SPHC1134/2012/CHN |
| KT033905 | DenHaag2006b | Hu/GII.4/DenHaag2006b/SPHC1183/2012/CHN |
| LC005707 | DenHaag2006b | Hu/GII.4/DenHaag2006b/y07-V203-1/2008/JP |
| LC005716 | DenHaag2006b | Hu/GII.4/DenHaag2006b/y11-V664-1/2012/JP |
| LC005718 | DenHaag2006b | Hu/GII.4/DenHaag2006b/y12-V758-3/2012/JP |
| LC005719 | DenHaag2006b | Hu/GII.4/DenHaag2006b/y12-V836-3/2012/JP |
| AB434770 | Osaka2007 | Hu/GII.4/Osaka2007/OC07138/2007/JP |
| AB541321 | Osaka2007 | Hu/GII.4/Osaka2007/Osaka2/2007/JP |
| EU876882 | Osaka2007 | Hu/GII.4/Osaka2007/Cairo2/2006/EGY |
| EU876884 | Osaka2007 | Hu/GII.4/Osaka2007/Cairo4/2006/EGY |
| EU876888 | Osaka2007 | Hu/GII.4/Osaka2007/Cairo8/2007/EGY |
| FJ411171 | Osaka2007 | Hu/GII.4/Osaka2007/SSCS/2005/USA |
| GQ246791 | Osaka2007 | Hu/GII.4/Osaka2007/Dijon/E3020/2008/FRA |
| GQ246800 | Osaka2007 | Hu/GII.4/Osaka2007/Dijon/E3880/2009/FRA |
| GQ413969 | Osaka2007 | Hu/GII.4/Osaka2007/Riviera1635/2008/US |
| GQ845368 | Osaka2007 | Hu/GII.4/Osaka2007/Sutherland/NSW505G/2007/AUS |
| GQ850882 | Osaka2007 | Hu/GII.4/Osaka2007/Riviera1590/2008/US |
| AB492092 | Apeldoorn2007 | Hu/GII.4/Apeldoorn2007/Stockholm/19865/2008/SE |
| AB541310 | Apeldoorn2007 | Hu/GII.4/Apeldoorn2007/Niigata1/2008/JP |
| AB541320 | Apeldoorn2007 | Hu/GII.4/Apeldoorn2007/Osaka1/2008/JP |
| AB933730 | Apeldoorn2007 | Hu/GII.4/Apeldoorn2007/Ehime1/2009/JP |
| GQ246792 | Apeldoorn2007 | Hu/GII.4/Apeldoorn2007/Dijon/2008/FRA |
| GQ246794 | Apeldoorn2007 | Hu/GII.4/Apeldoorn2007/Dijon/2008/FRA |
| GQ246798 | Apeldoorn2007 | Hu/GII.4/Apeldoorn2007/Dijon/2009/FRA |
| GQ303445 | Apeldoorn2007 | Hu/GII.4/Apeldoorn2007/Mannheim131/2009/DE |
| GU270580 | Apeldoorn2007 | Hu/GII.4/Apeldoorn2007/New_Orleans1500/2008/USA |
| GU390901 | Apeldoorn2007 | Hu/GII.4/Apeldoorn2007/Chungnam/2008/Kor |
| HM635100 | Apeldoorn2007 | Hu/GII.4/Apeldoorn2007/Seoul/2009/KOR |
| HM635101 | Apeldoorn2007 | Hu/GII.4/Apeldoorn2007/Seoul/2009/KOR |
| HM635155 | Apeldoorn2007 | Hu/GII.4/Apeldoorn2007/Seoul/2009/KOR |
| HQ005292 | Apeldoorn2007 | Hu/GII.4/Apeldoorn2007/2221223/2009/HK |
| HQ009513 | Apeldoorn2007 | Hu/GII.4/Apeldoorn2007/JB-15/2008/KOR |
| JQ613529 | Apeldoorn2007 | Hu/GII.4/Apeldoorn2007/NSW963U/2009/AU |
| JX445161 | Apeldoorn2007 | Hu/GII.4/Apeldoorn2007/AlbertaEI210/2008/CA |
| JX448566 | Apeldoorn2007 | Hu/GII.4/Apeldoorn2007/Seoul/2010/KOR |
| JX459630 | Apeldoorn2007 | Hu/GII.4/Apeldoorn2007/SG4079-07/2008/SG |
| JX459638 | Apeldoorn2007 | Hu/GII.4/Apeldoorn2007/SG4023-05/2009/SG |
| JX459903 | Apeldoorn2007 | Hu/GII.4/Apeldoorn2007/Jannali/2011/AU |
| KC409311 | Apeldoorn2007 | Hu/GII.4/Apeldoorn2007/30199/2009/VNM |
| KF429782 | Apeldoorn2007 | Hu/GII.4/Apeldoorn2007/NIHIC1.3/2010/USA |

Table S1 (continued). Strains used in this study (GII.4 genotype)

| GenBank  Accession No. | GII.4 variants | Names |
| --- | --- | --- |
| KF429791 | Apeldoorn2007 | Hu/GII.4/Apeldoorn2007/NIHIC1.5/2011/USA |
| KF712507 | Apeldoorn2007 | Hu/GII.4/Apeldoorn2007/NIHIC1.11/2012/USA |
| AB629944 | NewOrleans2009 | Hu/GII.4/NewOrleans2009/Tokyo/10-1443/2010/JPN |
| AB933741 | NewOrleans2009 | Hu/GII.4/NewOrleans2009/Chiba4/2009/JP |
| AB933745 | NewOrleans2009 | Hu/GII.4/NewOrleans2009/Aichi3/2010/JP |
| AB933746 | NewOrleans2009 | Hu/GII.4/NewOrleans2009/Osaka1/2009/JP |
| AB933748 | NewOrleans2009 | Hu/GII.4/NewOrleans2009/Ehime5/2011/JP |
| AB933753 | NewOrleans2009 | Hu/GII.4/NewOrleans2009/Ehime4/2009/JP |
| AB933754 | NewOrleans2009 | Hu/GII.4/NewOrleans2009/Saga1/2009/JP |
| AB933755 | NewOrleans2009 | Hu/GII.4/NewOrleans2009/Saga2/2009/JP |
| AB933769 | NewOrleans2009 | Hu/GII.4/NewOrleans2009/Nagano4/2011/JP |
| AB933771 | NewOrleans2009 | Hu/GII.4/NewOrleans2009/Hiroshimacity1/2011/JP |
| GQ845345 | NewOrleans2009 | Hu/GII.4/NewOrleans2009/NSW806J/2008/AU |
| HF952120 | NewOrleans2009 | Hu/GII.4/NewOrleans2009/C00007876/2011/UK |
| HF952122 | NewOrleans2009 | Hu/GII.4/NewOrleans2009/C00007880/2011/UK |
| HF952134 | NewOrleans2009 | Hu/GII.4/NewOrleans2009/C00007941/2011/UK |
| HM191773 | NewOrleans2009 | Hu/GII.4/NewOrleans2009/2200661/2010/HK |
| HM625866 | NewOrleans2009 | Hu/GII.4/NewOrleans2009/Pecs/HUN4322/2010/HUN |
| JN400622 | NewOrleans2009 | Hu/GII.4/NewOrleans2009/CGMH24/2010/TW |
| JN595867 | NewOrleans2009 | Hu/GII.4/NewOrleans2009/New_Orleans/2010/USA |
| JQ613539 | NewOrleans2009 | Hu/GII.4/NewOrleans2009/NSW447T/2009/AU |
| JQ613543 | NewOrleans2009 | Hu/GII.4/NewOrleans2009/NSW0159/2010/AU |
| JQ613553 | NewOrleans2009 | Hu/GII.4/NewOrleans2009/NSW186M/2010/AU |
| JQ613559 | NewOrleans2009 | Hu/GII.4/NewOrleans2009/NSW2004/2010/AU |
| JQ613561 | NewOrleans2009 | Hu/GII.4/NewOrleans2009/NSW234D/2010/AU |
| JQ613564 | NewOrleans2009 | Hu/GII.4/NewOrleans2009/NSW944J/2010/AU |
| JQ613573 | NewOrleans2009 | Hu/GII.4/NewOrleans2009/Helensburgh/NSW295E/2010/AU |
| JX439821 | NewOrleans2009 | Hu/GII.4/NewOrleans2009/Seoul1086/2010/KOR |
| JX439833 | NewOrleans2009 | Hu/GII.4/NewOrleans2009/Seoul1409/2010/KOR |
| JX445166 | NewOrleans2009 | Hu/GII.4/NewOrleans2009/AlbertaEI204/2010/CA |
| JX459653 | NewOrleans2009 | Hu/GII.4/NewOrleans2009/SG4014-04/2010/SG |
| JX459654 | NewOrleans2009 | Hu/GII.4/NewOrleans2009/SG4051-08/2010/SG |
| JX459656 | NewOrleans2009 | Hu/GII.4/NewOrleans2009/SG4054-08/2010/SG |
| JX459659 | NewOrleans2009 | Hu/GII.4/NewOrleans2009/SG4010-01/2011/SG |
| JX644038 | NewOrleans2009 | Hu/GII.4/NewOrleans2009/N100/2011/HuZhou |
| JX846928 | NewOrleans2009 | Hu/GII.4/NewOrleans2009/NIHIC9/2011/USA |
| JX984950 | NewOrleans2009 | Hu/GII.4/NewOrleans2009/GZ2010-L87/Guangzhou/2011/CHN |
| KC175385 | NewOrleans2009 | Hu/GII.4/NewOrleans2009/10368/2010/VNM |
| KC409241 | NewOrleans2009 | Hu/GII.4/NewOrleans2009/10405/2010/VNM |
| KC463910 | NewOrleans2009 | Hu/GII.4/NewOrleans2009/Ohio/684/2012/USA |
| KC577174 | NewOrleans2009 | Hu/GII.4/NewOrleans2009/Jiangsu1/2011/CHN |
| KC792279 | NewOrleans2009 | Hu/GII.4/NewOrleans2009/kaohsiung/12-AY-1/2010/TW |
| KF059996 | NewOrleans2009 | Hu/GII.4/NewOrleans2009/ACT0675/2012/AU |
| KF060006 | NewOrleans2009 | Hu/GII.4/NewOrleans2009/NSW097K/2012/AU |
| KF060013 | NewOrleans2009 | Hu/GII.4/NewOrleans2009/NSW1560/2011/AU |
| KF060014 | NewOrleans2009 | Hu/GII.4/NewOrleans2009/NSW1627/2012/AU |
| KF060016 | NewOrleans2009 | Hu/GII.4/NewOrleans2009/NSW1749/2011/AU |
| KF060018 | NewOrleans2009 | Hu/GII.4/NewOrleans2009/NSW2024/2012/AU |
| KF060025 | NewOrleans2009 | Hu/GII.4/NewOrleans2009/NSW270F/2012/AU |
| KF060048 | NewOrleans2009 | Hu/GII.4/NewOrleans2009/NSW4276/2011/AU |
| KF060049 | NewOrleans2009 | Hu/GII.4/NewOrleans2009/NSW431D/2011/AU |
| KF060055 | NewOrleans2009 | Hu/GII.4/NewOrleans2009/NSW468J/2011/AU |
| KF060071 | NewOrleans2009 | Hu/GII.4/NewOrleans2009/NSW590J/2012/AU |
| KF060072 | NewOrleans2009 | Hu/GII.4/NewOrleans2009/NSW596M/2011/AU |
| KF060078 | NewOrleans2009 | Hu/GII.4/NewOrleans2009/NSW619D/2012/AU |
| KF060079 | NewOrleans2009 | Hu/GII.4/NewOrleans2009/NSW623B/2011/AU |
| KF060093 | NewOrleans2009 | Hu/GII.4/NewOrleans2009/NSW7117/2012/AU |
| KF060099 | NewOrleans2009 | Hu/GII.4/NewOrleans2009/NSW783I/2011/AU |

Table S1 (continued). Strains used in this study (GII.4 genotype)

| GenBank  Accession No. | GII.4 variants | Names |
| --- | --- | --- |
| KF060115 | NewOrleans2009 | Hu/GII.4/NewOrleans2009/NSW906P/2012/AU |
| KF060119 | NewOrleans2009 | Hu/GII.4/NewOrleans2009/NSW9393/2012/AU |
| KF429778 | NewOrleans2009 | Hu/GII.4/NewOrleans2009/NIHIC18.1/2012/USA |
| KF768471 | NewOrleans2009 | Hu/GII.4/NewOrleans2009/P3/Gothenburg/2012/Sweden |
| KJ407073 | NewOrleans2009 | Hu/GII.4/NewOrleans2009/HS292/2012/USA |
| KJ685403 | NewOrleans2009 | Hu/GII.4/NewOrleans2009/BG1C0204/2011/BGD |
| KJ685405 | NewOrleans2009 | Hu/GII.4/NewOrleans2009/BG1C0282/2011/BGD |
| KJ685408 | NewOrleans2009 | Hu/GII.4/NewOrleans2009/BG1C0066/2011/BGD |
| KM198544 | NewOrleans2009 | Hu/GII.4/NewOrleans2009/C2418/2010/VNM |
| KP244314 | NewOrleans2009 | Hu/GII.4/NewOrleans2009/PR11471/2011/ITA |
| KP244315 | NewOrleans2009 | Hu/GII.4/NewOrleans2009/PR7841/2011/ITA |
| KP244316 | NewOrleans2009 | Hu/GII.4/NewOrleans2009/PR9474/2011/ITA |
| KP244317 | NewOrleans2009 | Hu/GII.4/NewOrleans2009/PR328/2013/ITA |
| KP244318 | NewOrleans2009 | Hu/GII.4/NewOrleans2009/PA288/2011/ITA |
| KP244320 | NewOrleans2009 | Hu/GII.4/NewOrleans2009/PA330/2011/ITA |
| KP244322 | NewOrleans2009 | Hu/GII.4/NewOrleans2009/PA99/2012/ITA |
| KR904207 | NewOrleans2009 | Hu/GII.4/NewOrleans2009/Johannesburg_3440/2009/ZA |
| KR904209 | NewOrleans2009 | Hu/GII.4/NewOrleans2009/Johannesburg_3611/2009/ZA |
| KR904210 | NewOrleans2009 | Hu/GII.4/NewOrleans2009/Johannesburg_4019/2009/ZA |
| KR904212 | NewOrleans2009 | Hu/GII.4/NewOrleans2009/Bushbuckridge_5246/2010/ZA |
| KR904213 | NewOrleans2009 | Hu/GII.4/NewOrleans2009/Johannesburg_5454/2010/ZA |
| KR904216 | NewOrleans2009 | Hu/GII.4/NewOrleans2009/Johannesburg_6298/2010/ZA |
| KR904217 | NewOrleans2009 | Hu/GII.4/NewOrleans2009/Empangeni_6336/2010/ZA |
| KR904218 | NewOrleans2009 | Hu/GII.4/NewOrleans2009/Pietermaritzburg_6370/2010/ZA |
| KR904220 | NewOrleans2009 | Hu/GII.4/NewOrleans2009/Cape_Town_10103/2012/ZA |
| KR904225 | NewOrleans2009 | Hu/GII.4/NewOrleans2009/Johannesburg_12232/2013/ZA |
| KR904234 | NewOrleans2009 | Hu/GII.4/NewOrleans2009/Empangeni_7470/2011/ZA |
| KR904235 | NewOrleans2009 | Hu/GII.4/NewOrleans2009/Bushbuckridge_8108/2011/ZA |
| KT033903 | NewOrleans2009 | Hu/GII.4/NewOrleans2009/SPHC2715/2012/CHN |
| LN854571 | NewOrleans2009 | Hu/GII.4/NewOrleans2009//Nijmegen01/2012/NL |
| KC456070 | Sydney2012 | Hu/GII.4/Sydney2012/VP1172/Shanghai/2012/CHN |
| KC456071 | Sydney2012 | Hu/GII.4/Sydney2012/VP1214/Shanghai/2012/CHN |
| KC456072 | Sydney2012 | Hu/GII.4/Sydney2012/VP1281/Shanghai/2012/CHN |
| KC517362 | Sydney2012 | Hu/GII.4/Sydney2012/Taoyuan/CGMH52/2012/TW |
| KC517370 | Sydney2012 | Hu/GII.4/Sydney2012/Taoyuan/CGMH60/2012/TW |
| KF008241 | Sydney2012 | Hu/GII.4/Sydney2012/Paris-E8509/2012/FRA |
| KF008242 | Sydney2012 | Hu/GII.4/Sydney2012/Cuers-E8549/2012/FRA |
| KF060009 | Sydney2012 | Hu/GII.4/Sydney2012/NSW114H/2012/AU |
| KF060041 | Sydney2012 | Hu/GII.4/Sydney2012/NSW369N/2012/AU |
| KF060046 | Sydney2012 | Hu/GII.4/Sydney2012/NSW426E/2012/AU |
| KF060062 | Sydney2012 | Hu/GII.4/Sydney2012/NSW558V/2012/AU |
| KF060080 | Sydney2012 | Hu/GII.4/Sydney2012/NSW628G/2012/AU |
| KF060112 | Sydney2012 | Hu/GII.4/Sydney2012/NSW8632/2012/AU |
| KF060129 | Sydney2012 | Hu/GII.4/Sydney2012/NLV-12-308/2012/NZ |
| KF145148 | Sydney2012 | Hu/GII.4/Sydney2012/JP10909/2012/JPN |
| KF177440 | Sydney2012 | Hu/GII.4/Sydney2012/32973745/2012/AUS |
| KF177446 | Sydney2012 | Hu/GII.4/Sydney2012/90186710/2012/AUS |
| KF177448 | Sydney2012 | Hu/GII.4/Sydney2012/35408489/2012/AUS |
| KF306214 | Sydney2012 | Hu/GII.4/Sydney2012/Jingzhou/2013/CHN |
| KF378731 | Sydney2012 | Hu/GII.4/Sydney2012/PA13/2013/ITA |
| KF668568 | Sydney2012 | Hu/GII.4/Sydney2012/PA48/2012/ITA |
| KJ433968 | Sydney2012 | Hu/GII.4/Sydney2012/12-AS-1/2012/Taiwan |
| KJ678141 | Sydney2012 | Hu/GII.4/Sydney2012/Beijing/PKUPH-02-08/2013/CHN |
| KJ678152 | Sydney2012 | Hu/GII.4/Sydney2012/Beijing/PKUPH-07-03/2013/CHN |
| KJ685402 | Sydney2012 | Hu/GII.4/Sydney2012//BG1C0434/2012/BGD |
| KJ710247 | Sydney2012 | Hu/GII.4/Sydney2012/Johannesburg9814/2012/ZA |
| KJ716358 | Sydney2012 | Hu/GII.4/Sydney2012/Beijing/PKUPH-40/2012/CHN |
| KJ955493 | Sydney2012 | Hu/GII.4/Sydney2012/JB031230049/Nanshan/OB/2012/SZ/CHN |
| KM268097 | Sydney2012 | Hu/GII.4/Sydney2012/Hong_Kong/CUHK-NS-295/2014/CHN |
| KM268101 | Sydney2012 | Hu/GII.4/Sydney2012/Hong_Kong/CUHK-NS-299/2014/CHN |

Table S1 (continued). Strains used in this study (GII.4 genotype)

| GenBank  Accession No. | GII.4 variants | Names |
| --- | --- | --- |
| KM396961 | Sydney2012 | Hu/GII.4/Sydney2012/CUHK-NS-339/2014/HKG |
| KM514064 | Sydney2012 | Hu/GII.4/Sydney2012/CUHK-NS-319/2014/HKG |
| KM514075 | Sydney2012 | Hu/GII.4/Sydney2012/CUHK-NS-332/2014/HKG |
| KM982955 | Sydney2012 | Hu/GII.4/Sydney2012/CUHK-NS-369/2014/HKG |
| KP096330 | Sydney2012 | Hu/GII.4/Sydney2012/CUHK-NS-377/2014/HKG |
| KP096335 | Sydney2012 | Hu/GII.4/Sydney2012/CUHK-NS-385/2014/HKG |
| KP096338 | Sydney2012 | Hu/GII.4/Sydney2012/CUHK-NS-389/2014/HKG |
| KP096339 | Sydney2012 | Hu/GII.4/Sydney2012/CUHK-NS-393/2014/HKG |
| KP096342 | Sydney2012 | Hu/GII.4/Sydney2012/CUHK-NS-396/2014/HKG |
| KP096346 | Sydney2012 | Hu/GII.4/Sydney2012/CUHK-NS-402/2014/HKG |
| KP096348 | Sydney2012 | Hu/GII.4/Sydney2012/CUHK-NS-406/2014/HKG |
| KP176403 | Sydney2012 | Hu/GII.4/Sydney2012/CUHK-NS-416/2014/HKG |
| KP176411 | Sydney2012 | Hu/GII.4/Sydney2012/CUHK-NS-433/2014/HKG |
| KP241905 | Sydney2012 | Hu/GII.4/Sydney2012/CUHK-NS-436/2014/HKG |
| KP241906 | Sydney2012 | Hu/GII.4/Sydney2012/CUHK-NS-437/2014/HKG |
| KP241907 | Sydney2012 | Hu/GII.4/Sydney2012/CUHK-NS-439/2014/HKG |
| KP241911 | Sydney2012 | Hu/GII.4/Sydney2012/CUHK-NS-445/2014/HKG |
| KP241914 | Sydney2012 | Hu/GII.4/Sydney2012/CUHK-NS-453/2014/HKG |
| KP698923 | Sydney2012 | Hu/GII.4/Sydney2012/CUHK-NS-474/2014/HKG |
| KP698924 | Sydney2012 | Hu/GII.4/Sydney2012/CUHK-NS-485/2014/HKG |
| KP698926 | Sydney2012 | Hu/GII.4/Sydney2012/CUHK-NS-490/2014/HKG |
| KP864105 | Sydney2012 | Hu/GII.4/Sydney2012/142696/Shanghai/2014/CHN |
| KP864107 | Sydney2012 | Hu/GII.4/Sydney2012/152624/Shanghai/2015/CHN |
| KR904221 | Sydney2012 | Hu/GII.4/Sydney2012/Empangeni_11939/2013/ZA |
| KR904237 | Sydney2012 | Hu/GII.4/Sydney2012/Empangeni_8598/2012/ZA |
| KR904238 | Sydney2012 | Hu/GII.4/Sydney2012/Cape_Town_10917/2013/ZAF |
| KT780375 | Sydney2012 | Hu/GII.4/Sydney2012/CUHK-NS-525/2015/HKG |
| KT780381 | Sydney2012 | Hu/GII.4/Sydney2012/CUHK-NS-554/2015/HKG |
| KT780388 | Sydney2012 | Hu/GII.4/Sydney2012/CUHK-NS-618/2015/HKG |
| KT780389 | Sydney2012 | Hu/GII.4/Sydney2012/CUHK-NS-651/2015/HKG |
| KT780392 | Sydney2012 | Hu/GII.4/Sydney2012/CUHK-NS-661/2015/HKG |
| LC005720 | Sydney2012 | Hu/GII.4/Sydney2012/y11-V615-5/2011/JP |
| LC005727 | Sydney2012 | Hu/GII.4/Sydney2012/y12-V780-1/2012/JP |
| LC005730 | Sydney2012 | Hu/GII.4/Sydney2012/y12-V849-2/2013/JP |
| LC018706 | Sydney2012 | Hu/GII.4/Sydney2012/Aichi368-14/2014/JP |
| LC018709 | Sydney2012 | Hu/GII.4/Sydney2012/Aichi352-14/2014/JP |
| LN854566 | Sydney2012 | Hu/GII.4/Sydney2012/Groningen01/2014/NL |
| LN854567 | Sydney2012 | Hu/GII.4/Sydney2012/Groningen02/2014/NL |
| AB684675 | Not typed | Hu/GII.4/21-5/Tokyo/1975/JPN |
| AB684704 | Not typed | Hu/GII.4/52-2/Tokyo/1980/JPN |
| AB684705 | Not typed | Hu/GII.4/53-1/Tokyo/1980/JPN |
| AB684720 | Not typed | Hu/GII.4/64-3/Tokyo/1983/JPN |
| FJ537135 | Not typed | Hu/GII.4/CHDC2094/1974/US |
| FJ537138 | Not typed | Hu/GII.4/CHDC4871/1977/US |
| HQ008055 | Not typed | Hu/GII.4/8483/2008/ZAF |
| JX023286 | Not typed | Hu/GII.4/CHDC5191/1974/USA |
| JX401279 | Not typed | Hu/GII.4/C127/1978/GF |
| JX401280 | Not typed | Hu/GII.4/KL45/1978/MY |
| JX401281 | Not typed | Hu/GII.4/T091/1976/TN |
| KC962453 | Not typed | Hu/GII.4/Bushbuckridge/5928/2010/ZAF |
| KF429760 | Not typed | Hu/GII.4/NIHIC28.4/2012/USA |
| KJ710245 | Not typed | Hu/GII.4/6745/CapeTown/2011/ZAF |

| Domains | Shell | P1 | P2 |
| --- | --- | --- | --- |
| Shell |  | < 2E-16 | < 2E-16 |
| P1 | *** |  | < 2E-16 |
| P2 | *** | *** |  |

Table S2. Statistical analyses with the multiple comparisons for evolutionary rates in the domains of GII.4 *VP1* gene

The *p*-values and significant signs were shown in the upper and lower sides, respectively.

*** *p* < 0.001

Table S3. Statistical analyses with the multiple comparisons for evolutionary rates in the present GII.4 variant strains

| GII.4 variants | US95_96 | Farmington Hills 2002 | Asia 2003 | Hunter 2004 | Yerseke 2006a | Den Haag 2006b | Osaka 2007 | Apeldoorn 2007 | New Orleans 2009 | Sydney 2012 |
| --- | --- | --- | --- | --- | --- | --- | --- | --- | --- | --- |
| US95_96 |  | < 2E-16 | < 2E-16 | < 2E-16 | < 2E-16 | < 2E-16 | < 2E-16 | < 2E-16 | < 2E-16 | < 2E-16 |
| Farmington Hills 2002 | *** |  | < 2E-16 | < 2E-16 | < 2E-16 | < 2E-16 | < 2E-16 | < 2E-16 | < 2E-16 | < 2E-16 |
| Asia 2003 | *** | *** |  | < 2E-16 | < 2E-16 | < 2E-16 | < 2E-16 | < 2E-16 | < 2E-16 | < 2E-16 |
| Hunter 2004 | *** | *** | *** |  | < 2E-16 | < 2E-16 | < 2E-16 | < 2E-16 | < 2E-16 | < 2E-16 |
| Yerseke 2006a | *** | *** | *** | *** |  | < 2E-16 | < 2E-16 | < 2E-16 | < 2E-16 | < 2E-16 |
| Den Haag 2006b | *** | *** | *** | *** | *** |  | < 2E-16 | < 2E-16 | < 2E-16 | < 2E-16 |
| Osaka 2007 | *** | *** | *** | *** | *** | *** |  | < 2E-16 | < 2E-16 | < 2E-16 |
| Apeldoorn 2007 | *** | *** | *** | *** | *** | *** | *** |  | < 2E-16 | < 2E-16 |
| New Orleans 2009 | *** | *** | *** | *** | *** | *** | *** | *** |  | < 2E-16 |
| Sydney 2012 | *** | *** | *** | *** | *** | *** | *** | *** | *** |  |

The *p*-values and significant signs were shown in the upper and lower sides, respectively.

*** *p* < 0.001

Table S4. Statistical analyses with the multiple comparisons for phylogenetic distances in the present GII.4 variant strains

| GII.4 variants | Camberwell  1994 | US95_96 | Kaiso 2003 | Farmington  Hills 2002 | Lanzou  2002 | Asia  2003 | Hunter  2004 | Yerseke  2006a | Den Haag  2006b | Osaka  2007 | Apeldoorn  2007 | New Orleans  2009 | Sydney  2012 |
| --- | --- | --- | --- | --- | --- | --- | --- | --- | --- | --- | --- | --- | --- |
| Camberwell 1994 |  | 6.7E-1 | 6.1E-1 | 3.5E-3 | 7.5E-1 | 1.2E-3 | 1.8E-3 | 1.9E-3 | 1 | 1 | 1.2E-1 | 1 | 2.7E-2 |
| US95_96 |  |  | 1 | < 2E-16 | 1 | < 2E-16 | < 2E-16 | < 2E-16 | 7.1E-15 | 4.7E-11 | 2.1E-8 | 6.3E-14 | < 2E-16 |
| Kaiso 2003 |  |  |  | 1 | 1 | 1 | 1 | 1 | 6.9E-1 | 5.5E-1 | 1 | 6.7E-1 | 1 |
| Farmington Hills 2002 | ** | *** |  |  | 6.9E-1 | < 2E-16 | 1 | 4.0E-3 | < 2E-16 | 1.9E-14 | 1.5E-4 | < 2E-16 | 8.0E-7 |
| Lanzou 2002 |  |  |  |  |  | 1.8E-4 | 6.5E-1 | 4.5E-2 | 6.9E-1 | 3.0E-2 | 1 | 6.9E-1 | 1 |
| Asia 2003 | ** | *** |  | *** | *** |  | < 2E-16 | 6.0E-6 | < 2E-16 | < 2E-16 | < 2E-16 | < 2E-16 | < 2E-16 |
| Hunter 2004 | ** | *** |  |  |  | *** |  | 1.1E-4 | < 2E-16 | 7.7E-16 | 2.8E-5 | < 2E-16 | 2.9E-9 |
| Yerseke 2006a | ** | *** |  | ** | * | *** | *** |  | < 2E-16 | 4.4E-13 | 7.8E-11 | <2E-16 | 1.1E-14 |
| Den Haag 2006b |  | *** |  | *** |  | *** | *** | *** |  | 1.2E-8 | < 2E-16 | 1 | < 2E-16 |
| Osaka 2007 |  | *** |  | *** | * | *** | *** | *** | *** |  | 4.8E-11 | 1.3E-8 | 4.5E-16 |
| Apeldoorn 2007 |  | *** |  | *** |  | *** | *** | *** | *** | *** |  | < 2E-16 | 1 |
| New Orleans 2009 |  | *** |  | *** |  | *** | *** | *** |  | *** | *** |  | < 2E-16 |
| Sydney 2012 | * | *** |  | *** |  | *** | *** | *** | *** | *** |  | *** |  |

The *p*-values and significant signs were shown in the upper and lower sides, respectively.

* *p* <0.05, ** *p* < 0.01, *** *p* < 0.001
